# Association between Total Dietary Phytochemical Intake and Cardiometabolic Health Outcomes—Results from a 10-Year Follow-Up on a Middle-Aged Cohort Population

**DOI:** 10.3390/nu15224793

**Published:** 2023-11-15

**Authors:** Magda Gamba, Octavio Pano, Peter Francis Raguindin, Zayne M. Roa-Diaz, Taulant Muka, Marija Glisic, Oscar H. Franco, Pedro Marques-Vidal

**Affiliations:** 1 Institute of Social and Preventive Medicine (I.S.P.M.), University of Bern, 3012 Bern, Switzerland; 2 Graduate School for Health Sciences, University of Bern, 3012 Bern, Switzerland; 3 Navarra Institute for Health Research (IdiSNA), 31009 Pamplona, Spain; 4 Faculty of Health Sciences and Medicine, University of Lucerne, 6005 Lucerne, Switzerland; 5 Medical Library, University of Bern, 3012 Bern, Switzerland; 6 Instituto Proinapsa, Universidad Industrial de Santander, Bucaramanga 680002, Colombia; 7 Epistudia, 3008 Bern, Switzerland; 8 Swiss Paraplegic Research, 6207 Nottwil, Switzerland; 9 Julius Center for Health Sciences and Primary Care, University Medical Center Utrecht, 3508 GA Utrecht, The Netherlands; 10 Department of Medicine, Internal Medicine, Lausanne University Hospital (C.H.U.V.) and University of Lausanne, 1011 Lausanne, Switzerland; pedro-manuel.marques-vidal@chuv.ch

**Keywords:** Dietary Phytochemical Index, Healthy Plant-Based Diet Index, phytochemical-rich foods, cardiovascular disease incidence, all-cause mortality, prospective study

## Abstract

Dietary phytochemical intake associations with cardiovascular health and mortality remain unknown. We studied the relations between total dietary phytochemical intake and cardiovascular health outcomes in a middle-aged Swiss population. We analyzed data spanning 2009 to 2021 from a prospective cohort study in Lausanne, Switzerland, including 3721 participants (54.8% women, 57.2 ± 10.3 years) without cardiovascular disease (CVD) history. Dietary intake was assessed using a validated self-reported food frequency questionnaire. The Dietary Phytochemical Index (DPI) and the healthy Dietary Phytochemical Index (hDPI) were calculated as the total energy intake percentage obtained from phytochemical-rich food consumption. The Healthy Plant-Based Diet Index (hPBD) was estimated by scoring healthy plant foods positively and less-healthy plant foods negatively. Indices tertiles and cardiometabolic outcome associations were determined using Cox proportional hazard models. Over 30,217 person-years of follow-up, 262 CVD events, and 178 deaths occurred. Unadjusted analyses found 36%, 33%, and 32% lower CVD risk for the highest hDPI, DPI, and hPBD tertiles, respectively. After adjustment, only the second hDPI tertile showed a 30% lower CVD risk (HR 0.70, 95% CI 0.51–0.95; P for trend 0.362). No other associations emerged. In this middle-aged Swiss cohort, no associations between dietary indices reflecting a phytochemical-rich dietary pattern and incident CVD, all-cause, or CVD mortality were observed.

## 1. Introduction

Cardiovascular diseases (CVD) are the leading global cause of death, accounting for 20.5 million deaths annually [1]. In Europe, CVD affects an estimated 113 million people, making it the predominant cause of death in the region [2]. Among the behavioral factors contributing to CVD development, unhealthy and poor-quality diets are prominent [3]. The primary dietary risk factors for CVDs include deficiencies in whole grain consumption, elevated sodium intake, insufficient fruit consumption, a lack of nuts and seeds, and inadequate vegetable intake [4].

Plant-based diets (PBDs) are environmentally sustainable dietary patterns, mainly comprising vegetables, fruits, whole grains, nuts, and pulses [5]. PBDs can be measured via dietary indices and have been related to lower CVD incidence, CVD mortality, and all-cause mortality [6,7] and are currently recommended in most prevention guidelines [8,9]. One possible pathway mediating those relations is the high phytochemical content of plant foods, also known as phytochemical-rich foods (PRFs). These PRFs comprise whole grains, fruits, vegetables, legumes, nuts, seeds, olive oil, cocoa, tea, coffee, and alcoholic beverages like beer and wine. PRFs contain phytochemical compounds like polyphenols, alkaloids, organosulfur compounds, and terpenoids (e.g., carotenoids and phytosterols) [10]. Such plant compounds present a widely documented cardiovascular protective effect via modulation of risk factors [11,12,13,14].

However, focusing research on isolated bioactive compounds does not capture the synergistic effect of dietary patterns constituted by foods and their nutrients and phytochemicals on human health [15]. Moreover, measuring dietary phytochemical intake in large population-based studies is challenging as it requires linking dietary assessment tools like food frequency questionnaires (FFQs) to databases of phytochemical information. This linkage is not always possible due to data accuracy concerns, as many of these databases may be outdated or incomplete [16]. Thus, the Dietary Phytochemical Index (DPI) was proposed to assess the impact of total dietary phytochemical intake on health and reflects such dietary interactions [17]. The DPI determines the percentage of daily dietary calories derived from PRFs, encompassing vegetables, fruits, whole grains, legumes, nuts, seeds, extra-virgin olive oil, and alcoholic beverages such as wine, beer, and cider [18]. It is hypothesized that high DPI values could be associated with better health outcomes. 

DPI has been used to assess the impact of PRF diets on cardiometabolic health. Nevertheless, evidence has been mainly collected from Iranian and South Korean cross-sectional studies and short-term follow-up cohorts, with mixed results [17,19] warranting further research on other populations with different dietary phytochemical intakes to understand better the impact of PRFs on human health and expand the DPI generalizability across other contexts and populations [17]. Recently, we have used the DPI for the first time in a European population to assess its cross-sectional association with cardiometabolic risk factors and metabolic syndrome, finding inverse associations for waist circumference, body mass index, insulin, leptin, and hs C-reactive protein, and lower odds of central obesity [20]. Nevertheless, to our knowledge, no study has evaluated the association of the DPI with CVD incidence and mortality in a prospective setting.

Therefore, we aimed to study the association between the DPI and CVD incidence, CVD mortality, and all-cause mortality in a population-based cohort of middle-aged participants living in Switzerland. We hypothesized that higher DPI values would reduce the risk of CVD incidence, CVD mortality, and all-cause mortality.

## 2. Materials and Methods

### 2.1. Study Population

The CoLaus study is a population-based cohort designed to assess epidemiological and genetic determinants of cardiovascular risk factors in participants aged 35–75 in Lausanne, Switzerland [21]. Between June 2003 and May 2006, 6733 participants were enrolled and underwent a blood and physical exam and an interview. Information related to dietary intake and physical activity was collected from the first follow-up (from April 2009 to September 2012) onwards. Data from the first follow-up, serving as the baseline for this study and including 5064 of the initial participants, and the third follow-up (from April 2018 to May 2021) were used for this study. 

### 2.2. Cardiovascular Outcomes Ascertainment 

During the follow-up period, first-incident CVD events (including fatal and non-fatal cases) and deaths were prospectively collected and independently adjudicated according to established recommendations and similar definitions detailed elsewhere [22]. CVD composite comprised fatal and non-fatal cases of coronary artery disease (CAD), major coronary events, and stroke. Major coronary events included acute coronary syndromes (acute myocardial infarction (A.M.I.) or unstable angina) and symptomatic stable angina followed by a revascularization procedure (either by percutaneous coronary intervention or by coronary artery bypass grafting). CAD events corresponded to participants who presented with typical symptoms (stable angina) and underwent either percutaneous (PTCA ± stenting) or surgical revascularizations unless these procedures were directly related to an A.M.I. Stroke was defined as rapidly developing clinical signs of focal or global disturbance of cerebral function of presumed vascular origin lasting ≥24 h. Briefly, incident CVD cases were recorded through a stepwise process. First, medical records and encompassed medical and/or surgical notes, laboratory, radiological, echocardiographic, and electrocardiographic reports of participants declaring a CVD and/or CVD-related procedure were collected. Second, events that may not have been mentioned during interviews were retrieved by searching the medical database of the University Hospital of Lausanne, which is the main community hospital in the catchment area of the study; events of interest were detected and adjudicated by two cardiologists or one neurologist (for stroke) using the ICD-10 (International Classification of Diseases, Tenth Edition) [23]. Mortality was ascertained and classified as cardiovascular and non-cardiovascular by two internists using the population register of the city where the participant lived in case of returned mail, absence of response when calling, and/or indication from a relative. Information on cause of death was sequentially collected from (1) general practitioners; (2) a medical database of the hospital where the death occurred (either in Switzerland or abroad); (3) a database of the pre-hospital emergency care unit of the City of Lausanne; (4) a database of the University Centres of Forensic Medicine of Lausanne and Geneva; (5) official death certificates from the Swiss Federal Office of Statistics, or (6) verbal autopsy with close relatives, if all previous steps failed. Details of the adjudication procedure are provided in Annex S1.

### 2.3. Dietary Assessment

Dietary intake for the previous four weeks was assessed using a validated, self-administered, semi-quantitative FFQ, including portion size [24].

The FFQ included 97 food items accounting for more than 90% intake of calories, proteins, fat, carbohydrates, alcohol, vitamin D, retinol, and 85% of fiber, carotene, and iron. For each item, seven consumption frequencies were provided, ranging from “less than once during the last four weeks” to “2 or more times per day”, and participants indicated the average serving size (smaller, equal, or bigger) compared with a reference size. Intake frequency was multiplied by the nutrient composition of the specified portion size expressed in milliliters (for drinks) and grams (for other food items) to determine caloric intake, which was based on the French CIQUAL (Centre d’Information sur la QUalité des ALiments) food composition table [25]. 

### 2.4. Dietary Indices Construction

The main exposures were the DPI, a healthier version of the DPI (hDPI), and the healthy Plant-Based Diet index (hPBD). In the case of the DPI and the hDPI, high index values represent a higher total phytochemical intake, whereas a higher hPBD signals a higher adherence to a healthy plant-based diet. 

DPI was operationalized as the percentage of dietary calories derived from PRFs following McCarthy’s proposal [18]:

$$DPI=\frac{daily\;energy\;intake\;from\;phytochemical\;rich\;foods\;(kcal)}{total\;daily\;energy\;intake\;(kcal)}\;\text{×}\;100$$

PRF items available from FFQ formed the numerator of the index calculation and included whole grains, vegetables, fruits, olive oil (for cooking), and alcohol (Table S1). Although the hDPI was constructed similarly to the DPI, this index excluded alcohol items (beer and wine), as their intake has controversial health effects [26]. 

The hPBD, categorizing food groups as “healthy plant-based”, “less-healthy plant-based”, and “animal”, was adapted from its original version [27]. The original hPBD includes 18 food groups; we created 16 food groups according to our FFQ items’ availability: five “healthy plant-based” groups (whole grains, fruits, vegetables, vegetable oil, and tea and coffee); five “less-healthy plant-based” groups (bottled fruit juice, refined grains, potatoes, sugar-sweetened beverages, and sweets and desserts) and six “animal” groups (animal fat, dairy, eggs, fish or seafood, meat, and miscellaneous animal-based foods) (Table S1). Servings/day were calculated for each food group as follows: first, the total amount consumed per day for each food group was computed using the daily frequency of intake and portion size. Second, the number of servings/day was computed by dividing the total amount by the average serving size, as indicated in the Swiss Food Pyramid [28]. The index was constructed by adding the food servings belonging to each food group and determining the 16 groups’ energy-adjusted consumption using the residual method [29]. The obtained values were divided into quintiles, where healthy plant-based foods were assigned a positive score ranging from 1 to 5 (e.g., highest quintile scored five points), while less-healthy plant-based foods and animal foods were reversely scored (e.g., highest quintile scored one point). All groups’ scores were added to obtain the total hPBD value and divided into tertiles. 

We included the hPBD in our analysis as it has been extensively used in the literature to associate adherence to PBDs and CVD incidence and mortality [6,7]. The hPBD shares some similarities with the DPI and the hDPI: firstly, hPBD food groups categorized as “healthy” include whole grains, vegetables, fruits, and vegetable oils, which are the same used to construct the DPI; secondly, the hPBD does not include alcohol like the hDPI, and lastly, both the hPBD and the hDPI emphasize the quality and healthiness of plant-based foods consumed.

### 2.5. Covariates Assessment

Data on demographic characteristics and lifestyle information were collected using self-administered questionnaires at the first follow-up. Potential confounders considered in adjustment were age (continuous), sex, educational level (university, high school, apprenticeship, and mandatory), physical activity (assessed by questionnaire [30] and expressed as total minutes/day), smoking status (never, former, and current), alcohol intake category (non-drinker, low (1–6 drinks/week), moderate (7–13/week) and high (14+/week)), total caloric intake (kcal/day), dieting (being on a diet for health reasons: y/n), body mass index (B.M.I., continuous), type 2 diabetes (T2D, defined as fasting plasma glucose ≥7 mmol or presence of antidiabetic drug treatment), hypertension (defined for a systolic blood pressure ≥140 mm Hg or a diastolic blood pressure ≥90 mm Hg or presence of anti-hypertensive drug treatment), hypercholesterolemia (defined as LDL-cholesterol >3.0 mmol/L or presence of hypolipidemic drug treatment), and family history of CVD and T2D. 

### 2.6. Ethical Considerations

The institutional Ethics Committee of the University of Lausanne, which afterward became the Ethics Commission of Canton Vaud (www.cer-vd.ch), approved the baseline CoLaus study (reference 16/03). Approval was renewed for the first (reference 33/09) and the third (reference PB_2018-00040) follow-ups. Approval for the entire CoLaus|PsyCoLaus study was confirmed in 2021 (reference PB_2018-00038, 239/09). The full decisions of the CER-VD can be obtained from the authors upon request. This study was performed in agreement with the Helsinki Declaration and its former amendments and in accordance with the applicable Swiss legislation. All participants gave their signed informed consent before entering this study.

### 2.7. Inclusion and Exclusion Criteria

General inclusion criteria for entering the CoLaus cohort were written informed consent and willingness to participate in an interview, physical examination, and providing blood samples. Exclusion criteria applied to this study were (1) missing data on dietary information; (2) implausible total energy intake (<850 kcal/day and >4500 kcal/day); (3) prior history of CVD, including major coronary event, CAD, and stroke; and (4) no follow-up information.

### 2.8. Statistical Analysis

Participants were categorized by tertiles of the dietary indices (the DPI, the hDPI, and the hPBD). The baseline characteristics of participants were adjusted by age and sex using inverse probability weighting (I.P.W.). We summarized as means and standard deviations (S.D.) or medians and interquartile range (IQR) for continuous variables and as number of participants (percentage) for categorical variables. 

Multivariable Cox proportional hazard regression models were used to estimate hazard ratios (H.R.) and 95% confidence intervals (CI) for the associations of each index tertile and outcomes of interest, taking as reference category tertile one. Proportionality assumptions were tested by examining Schoenfeld’s residuals. Time was scaled using age (in years), and person-years were computed from baseline to the date of CVD event, date of death, or date of return of the last follow-up questionnaire, whichever occurred first. We incrementally adjusted our models to assess for confounding, namely (1) the crude model, (2) the age- and sex-adjusted model, (3) the additional education level, physical activity, smoking status, alcohol intake (except for the DPI), total caloric intake (only for the hPBD), B.M.I. and dieting-adjusted model, and (4) the model additionally adjusted by T2D and hypertension history, hypercholesterolemia prevalence, and family history of T2D (only for all-cause mortality and CVD mortality) and CVD. For each index, linear trends across tertiles were assessed by introducing a single continuous variable containing the median value of the corresponding tertile for each participant. Nelson–Aelen curves were plotted to describe the cumulative risk of each outcome; the curves were adjusted by age, sex, smoking status, physical activity, B.M.I., total caloric intake (only for the hPBD), hypertension history, and family history of CVD and T2D using I.P.W. 

Subgroups and sensitivity analyses were also performed. Characteristics between included and excluded participants were compared to assess selection bias using a chi-square or Student’s *t*-test. We performed median imputation for any missing values on covariates. We did subgroup analyses by age (40–60 years old and >60 years old), sex, smoking (never, former, current), B.M.I. (normal weight (<25 kg/m^2^), pre-obesity and obesity (≥25 kg/m^2^)), familiar history of CVD and prevalent T2D. Because non-CVD mortalities preempt CVD-related deaths, we fitted our Cox regression models with the two outcomes as “competing risks”. Lastly, non-linear associations were explored by fitting cubic splines with three randomly assigned knots and adjusted by covariates as mentioned. Statistical analyses were performed using Stata version 17.0 for Windows (Stata Corp, College Station, TX, USA), and statistical significance was established for a two-sided test with *p* < 0.05.

## 3. Results

### 3.1. Sample Characteristics across Tertiles of Dietary Indexes

**Figure 1:**
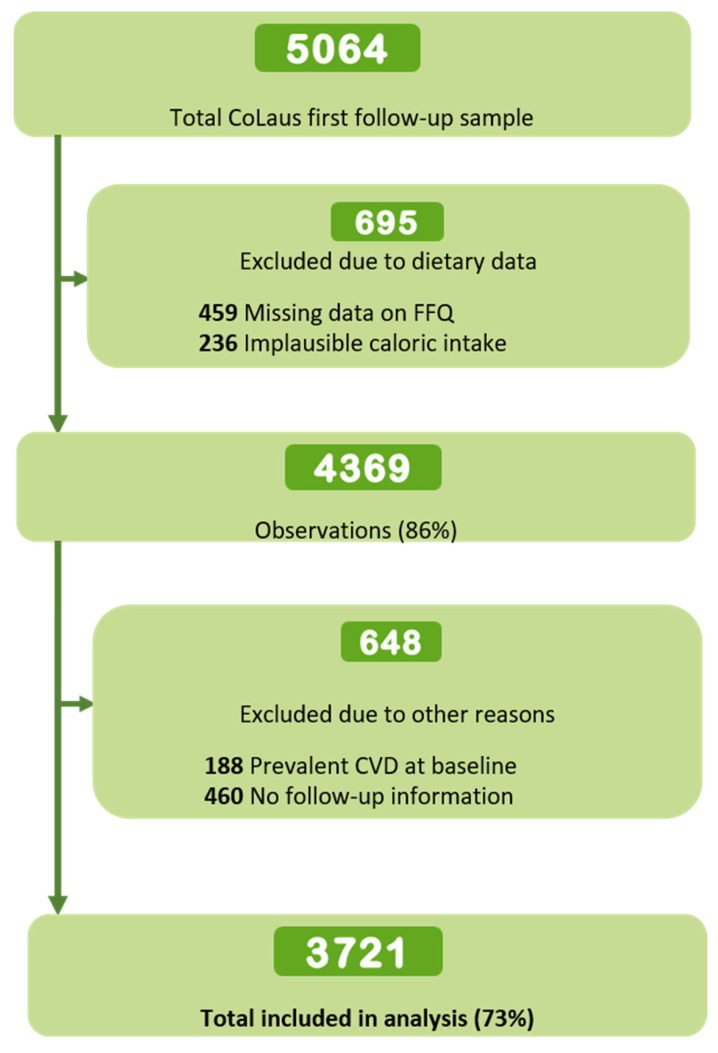
Flowchart of participants’ selection for the association between the hDPI, the DPI, and the hPBD and ten-year incidence of CVD, all-cause mortality, and CVD mortality. The CoLaus study, Lausanne, Switzerland, 2009–2012.

From the initial 5064 participants, 3721 (54.8% women, mean age 57.2 ± 10.3 years) were retained for analysis (Figure 1). Excluded participants had lower hDPI and hPBD means, were older, mostly men, had lower educational levels, smoked more, had a higher B.M.I., reported more family and personal history of disease (except for hypercholesterolemia), and used fewer dietary supplements. (Table S2). hDPI values ranged from 0 to 74.3%, with a median of 21.4% (IQR 14.1–31.1); DPI values ranged from 1.6 to 74.3%, with a median of 25.4% (IQR 17.7–34.6); hPBD values ranged from 29 to 74, with a median of 48 (IQR 43–53). Table 1 presents the sample’s main characteristics distributed by tertiles of each dietary index. In general, in the higher tertiles, participants tended to be older, mainly women, highly educated, more active, thinner (although they were overweight), less likely to consume alcohol, and to be current smokers. Participants with higher dietary indices also reported a higher proportion of hypertension (except for the hPBD), family history of CVD, and dieting. 

### 3.2. Dietary Intake across Tertiles of Dietary Indexes

Table S3 presents age and sex-adjusted food groups’ frequency intake across tertiles of dietary indexes at baseline. Total energy intake decreased from the first (T1) to the second (T2) tertile in the hDPI and the DPI and increased from T2 to T3 for the hPBD. In all dietary indexes, participants in T3 consumed higher quantities of PRFs (designated as “healthy food groups” for the hPBD). In contrast, the highest intakes of refined grains, potatoes, meat, and miscellaneous animal foods (designated as less-healthy and animal food groups for the hPBD) occurred in T1 for all indexes. The intake of PRF beverages like tea and coffee was higher among participants in T3 for the hDPI and the hPBD, while bottled fruit juice and sugar-sweetened beverages were less frequently consumed in this tertile for all indexes. A decline in alcohol intake per increased tertile was more evident for the hDPI and the hPBD, while the highest alcohol intake occurred in T2 for the DPI. No particular trends were observed for other food groups like sweets and desserts, animal fats, dairy, eggs, and fish and seafood. 

**Figure 2:**
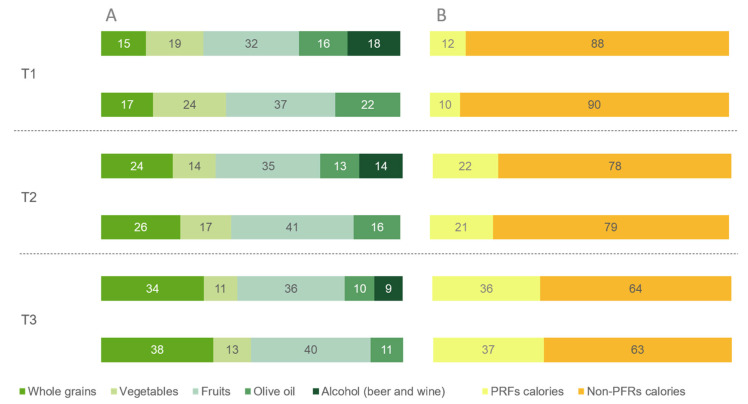
Phytochemical-rich foods’ caloric contribution according to the hDPI and the DPI tertiles. (**A**) food groups and (**B**) total caloric contribution. The CoLaus study, Lausanne, Switzerland, 2009–2012. Abbreviations: T, tertile. PRFs, phytochemical-rich foods. The hDPI excludes alcoholic beverages (beer and wine). Results are expressed as a percentage.

Figure 2A,B show the caloric contribution of PRFs to the hDPI and the DPI By food groups, fruits were the primary contributor in any of their tertiles, followed by vegetables in T1 and whole grains in T2 and T3. Conversely, the lowest caloric contributors for the hDPI and the DPI in T1 were whole grains, while olive oil was the lowest contributor in T2. As for T3, alcoholic beverages were the lowest contributors for the DPI and olive oil for the hDPI. As for the contribution to the total caloric intake, those in the higher tertiles of both indices had a threefold caloric intake from PRFs compared to participants in T1. 

### 3.3. Dietary Indices Associations with CVD Incidence

**Figure 3:**
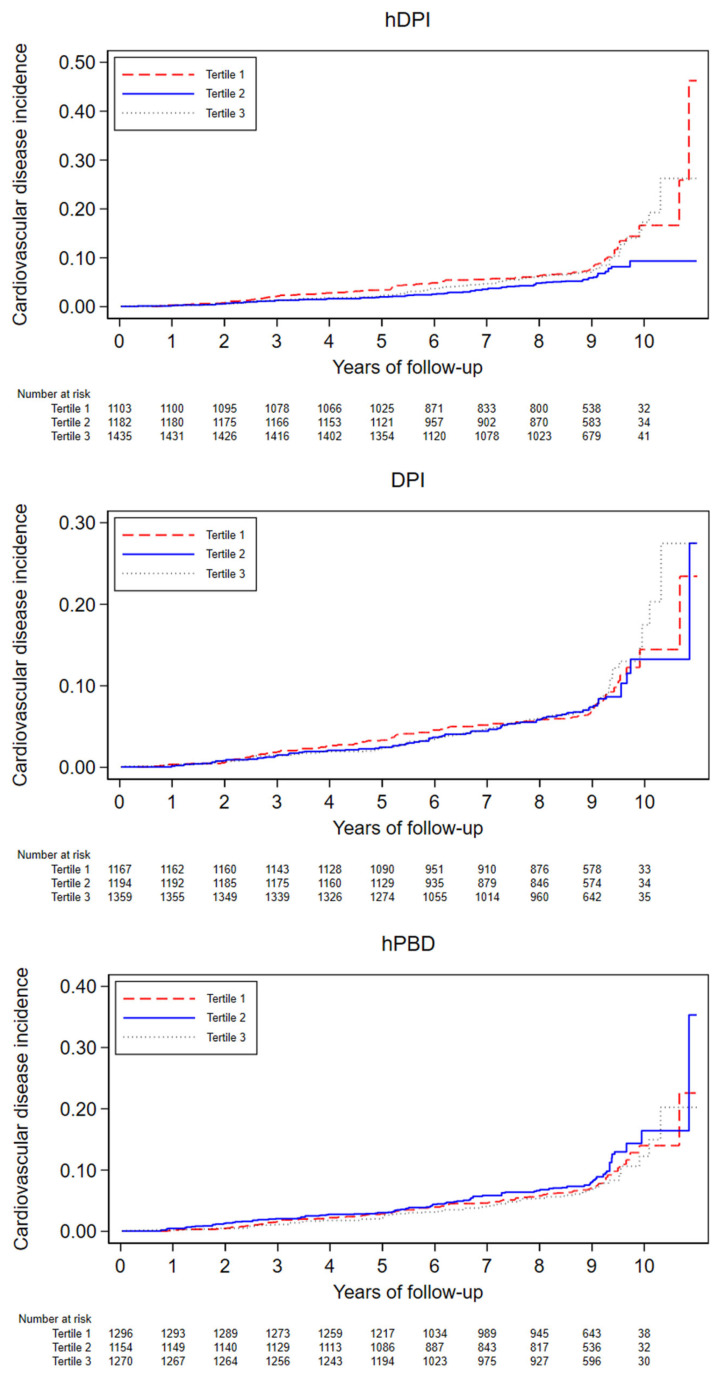
Adjusted Nelson–Aelen cumulative risk curves for CVD incidence. CoLaus study, Lausanne, Switzerland, 2009–2021. Curves are adjusted by age, sex, smoking status, physical activity, B.M.I., hypertension, and family history of CVD and T2D using I.P.W.

**Figure 4:**
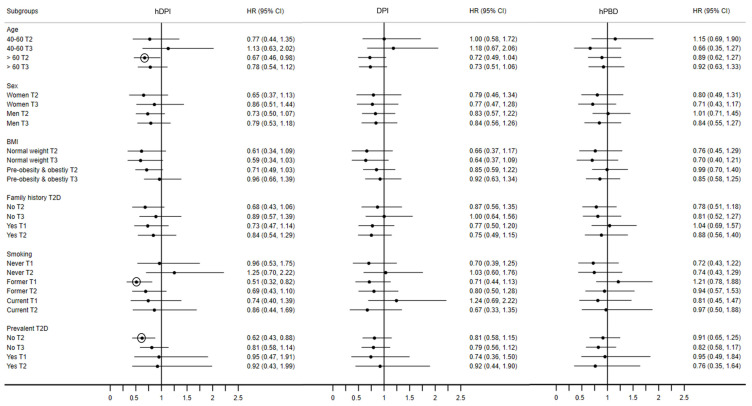
Hazard ratios (95% confidence interval) for the stratified analyses of multivariable associations between tertiles of the hDPI, the DPI, and the hPBD and incident cardiovascular disease (CVD). CoLaus study, Lausanne, Switzerland, 2009–2021. Abbreviations: T2, tertile 2. Tertile 3, T3. CVD, cardiovascular disease. T2D, Diabetes type 2. Black dots represent the estimates. Circles around the estimate denote a significant effect. All presented analyses estimated with fully adjusted models: age, sex, educational level, smoking status, alcohol consumption, physical activity, B.M.I., total caloric intake (only for the hPBD), dieting, T2D, hypertension, hypercholesterolemia and family history of CVD.

During a median of 9 years of follow-up and 30,217 person-years, there were 262 incident cases of CVD (incidence rate 8.6%). In unadjusted analysis, all indices had an inverse association with incident CVD (Table 2), but this association disappeared after multivariable adjustment, except for T2 of the hDPI. Those findings were further confirmed by adjusted Nelson–Aelen cumulative survival curves (Figure 3). Similarly, no significant associations between the DPI and the hPBD and incident CVD were found in stratified analyses (Figure 4 and Table S4). The protective effect of hDPI T2 remained for participants older than 60, former smokers, and participants without T2D. Finally, when assessing the associations using cubic splines (Figure S1), no evidence of non-linearity was found for any of the indexes when comparing fully adjusted linear vs. the cubic term models using likelihood ratio tests.

### 3.4. Dietary Indices Associations with CVD Mortality and All-Cause Mortality

After a median follow-up time of 9 years and 31,025 person-years, 178 participants died, 49 of whom related to CVD (incidence rates 5.7% and 1.5%, respectively). On bivariate analysis, a protective effect for hDPI T3 on all-cause mortality was observed, but this effect vanished after multivariable adjustment, while a deleterious effect of DPI T2 for CVD mortality appeared for the fully adjusted model (Table 3). The above overall lack of associations is also shown in adjusted Nelson–Aelen cumulative survival curves for all-cause and CVD mortality (Figure S2A,B, respectively).

### 3.5. Sensitivity Analyses

Observed results for all-cause mortality did not change in subgroup analyses (Table S5); due to the low number of CVD deaths, no stratified analyses were performed. When assessing the associations using cubic splines, no differences between models with linear and cubic terms for all-cause and CVD-related mortality were found (Figure S3A,B). Finally, no associations were observed between all dietary indexes and CVD mortality in competing risk analyses (Table S6).

## 4. Discussion

To our knowledge, this study is the first to assess the longitudinal association between indexes assessing total dietary phytochemical intake (the hDPI and the DPI) and incident CVD, CVD-related, and all-cause mortality. Besides a 30% lower risk of CVD incidence for participants in the second tertile of the hDPI, no other associations were observed. 

### 4.1. Dietary Indices Associations with CVD Incidence

Preliminary unadjusted findings indicated a one-third reduction in CVD risk among participants in the highest tertiles of the hDPI, the DPI, and the hPBD. Upon adjustment for relevant covariates, our results revealed no association between the indexes and CVD incidence, except for a reduced risk for participants in the second tertile of the hDPI across all adjustment models, suggesting a protective effect with a moderate dietary intake of phytochemicals. Nevertheless, the *p* value for trend did not reach statistical significance (*p*-trend = 0.362). 

Compared with existing literature on healthy dietary patterns, our results contradict current evidence, which usually reports inverse associations with CVD events [6,7,31,32]. One possible reason leading our findings towards a null result is the little variation observed among indices tertiles (e.g., the hDPI: median 21.4%, IQR 14.1–31.1). An additional plausible explanation for this finding might be related to the population itself; individuals in the third tertile were the oldest, suggesting other comorbidities that might interfere with phytochemical absorption, such as gastrointestinal disorders. We could not control for these comorbidities. Another issue is that DPI calculation considers that all ingested phytochemicals have a beneficial effect or that the higher the intake of phytochemicals, the more beneficial they are, which may not always be the case, as phytochemicals interactions can go from additive or synergistic to antagonistic [33], and therefore those interactions might modulate the health impact of total dietary phytochemical intake.

The only difference between the DPI and the hDPI derives from the exclusion in the hDPI of alcohol items, whose protective effect against CVD is being increasingly challenged [34,35]. The observed protective effect of hDPI T2 was not replicated when assessing the DPI. This finding might indicate that alcohol plays a detrimental role as part of total dietary phytochemical intake, and its intake would not contribute to dietary patterns aiming to prevent CVD. 

No association was found between hPBD and CVD incidence. These findings do not align with two recent meta-analyses, each including more than 400,000 participants and indicating that the hPBD is associated with a lower risk of CVD [6,7]. Still, our results replicate those of the Jackson Heart Study (J.H.S.) [36], which was conducted in a sample size similar to ours (*n* = 3635), in a slightly younger population (53.8 ± 12.5 years), with a longer follow-up (13 years), and a higher number of CVD events (293 vs. 262). This study also found no association between hPBD and CVD incidence, and the authors mentioned low quality and variability of the diet and a small number of cases as possible reasons for their results. Interestingly, participants in the CoLaus study also have a relatively low-quality diet, as in 2018, it was reported that almost two-thirds never complied with at least three guidelines from the Swiss Society of Nutrition [37]. Overall, it is possible that the lack of association between the dietary indexes and incident CVD events is due to a rather population presenting low compliance with dietary guidelines and/or low statistical power.

### 4.2. Dietary Indices Associations with CVD Mortality and All-Cause Mortality

Regarding all-cause mortality and CVD mortality, our results indicated, in general, no associations with evaluated dietary indices. The relation between the hPBD and mortality is controversial: two longitudinal studies, one including 13,000 Chinese individuals older than 65 years old and the other with 315,000 American adults, reported lower cardiovascular deaths with high hPBD scores [38,39], while the J.H.S. and a South-Korean cohort including 118,577 adults between 40 and 69 years old, found no association between the hPBD and all-cause and CVD mortality [36,40]. Finally, similar results were reported by a meta-analysis finding no associations between the hPBD and CVD mortality [6].

### 4.3. Strengths and Limitations

Our study is the first one assessing the impact of total dietary phytochemical intake on incident CVD, CVD-related and all-cause mortality using the DPI and its modified healthier version. It becomes part of the few longitudinal studies on the effect of total dietary phytochemical intake via the DPI on cardiovascular health [41,42,43]. We incrementally adjusted our models to account for important covariates, which is particularly interesting for the case of hDPI T2, where results remained robust across all models. Our analyses found no added value to the DPI by including phytochemicals originating from alcoholic beverages. 

Our study has the following limitations. First, measurement error leading to non-differential misclassification of participants might exist as dietary intake was measured using an FFQ, yet this questionnaire was validated on the Swiss–French population [24] and is the current gold standard in nutritional epidemiology [44]. Second, the sample was drawn from the French-speaking part of Switzerland and might not represent other Swiss cultural regions [45]. Third, compared with other prospective studies reporting inverse associations between the hPBD and cardiovascular health outcomes [27,46,47], our sample size is small (3721), and a low number of cases occurred (262). Hence, we might have low statistical power to detect associations, not only for the hPBD but also for the hDPI and the DPI. Lastly, residual confounding can not be ruled out given the nature of observational studies; we controlled for several lifestyles and CVD risk factors, but aspects like inter-individual variability related to differences in the bioavailability of phytochemicals and the variability in the phytochemical content of plant foods (attributable to factors like seasonality, crop varieties, and preservation) are important considerations; however, they are yet difficult to measure and account for in nutritional epidemiology.

### 4.4. Public Health Impact and Future Research

Applying the DPI to this Swiss population showed that the PRF groups that contribute the most to dietary phytochemical intake were fruits and whole grains. However, their intake was low among the CoLaus cohort participants; for instance, fruit intake is around 40% of the recommended amount for the Swiss population [37]. In this regard, higher efforts to promote whole grains and fruit intake among the Swiss population are encouraged. Several strategies to increase population intake should cover the entire production and distribution chain, better marketing of healthy plant-based foods, and include nutritional education and health literacy. 

Additional research on better understanding how total dietary phytochemical intake and adherence to PBDs are related to cardiovascular health is warranted, given the need to support dietary patterns that contribute to minimizing CVD burden while being sustainable [48]. Replication of longitudinal results in other populations and cohorts with larger sample sizes is required to correctly determine if our findings were hampered due to limited statistical power. As the DPI has been applied in more than 30 studies related to cardiometabolic health during the last decade, its validation is essential to improve results reliability and to determine cut-off values that clinicians can employ to guide patients to improve their phytochemical intake taking advantage of the fact that the DPI can be easily estimated. Future research in populations where alcohol intake is frequent should consider applying the healthy version of the DPI, as current research with this version of the index has shown robust results and, therefore, no need for including these items. 

## 5. Conclusions

In summary, our decade-long study of this cohort of middle-aged Swiss adults found that total dietary phytochemical intake (assessed via the hDPI and the DPI) and adherence to PBDs rich in phytochemicals (using the hPBD) were associated with a lower risk of CVD incidence in unadjusted analysis. However, these associations disappeared upon rigorous adjustments, and no significant associations were observed concerning CVD incidence, all-cause mortality, or CVD-related mortality. Further prospective studies with larger sample sizes are needed to confirm or refute these findings.

## Figures and Tables

**Table 1:** Baseline characteristics of participants across tertiles of the hDPI, the DPI, and the hPBD. The CoLaus study, Lausanne, Switzerland, 2009–2012.

	Healthy Dietary Phytochemical Index (hDPI)			Dietary Phytochemical Index (DPI)			Healthy Plant-Based Diet Index (hPBD)		
	Tertile 1	Tertile 2	Tertile 3	Tertile 1	Tertile 2	Tertile 3	Tertile 1	Tertile 2	Tertile 3
Number of participants	1127	1200	1394	1184	1212	1325	1371	1209	1141
Index median (IQR)	11 (8–14)	21 (19–24)	36 (31–43)	15 (12–18)	26 (23–28)	39 (35–46)	42 (39–44)	48 (47–50)	56 (53–59)
Age, mean (SD)	55.2 (10.2)	56.5 (10.2)	59.2 (10.2)	55.2 (10.4)	57.1 (10.2)	59.3 (10.0)	55.7 (10.4)	57.6 (10.4)	58.6 (9.9)
Sex, *n* (% women)	454 (37.4)	694 (55.5)	892 (70.7)	531 (42.8)	687 (55.2)	822 (66.3)	611 (43.7)	704 (57.7)	725 (65.7)
Education attainment, *n* (%)									
University education	246 (21.8)	231 (19.2)	319 (22.9)	258 (21.8)	232 (19.1)	321 (24.2)	329 (24.0)	233 (19.3)	281 (24.6)
High school	293 (26.0)	327 (27.2)	399 (28.6)	317 (26.8)	330 (27.2)	375 (28.3)	361 (26.3)	333 (27.5)	336 (29.4)
Apprenticeship	420 (37.3)	449 (37.4)	482 (34.6)	433 (36.6)	461 (38.1)	446 (33.7)	509 (37.1)	443 (36.7)	360 (31.6)
Mandatory education	168 (14.9)	194 (16.1)	194 (13.9)	176 (14.9)	189 (15.6)	184 (13.9)	172 (12.6)	200 (16.5)	164 (14.3)
Smoking status, *n* (%)									
Never	430 (38.1)	521 (43.4)	675 (48.4)	502 (42.4)	523 (43.2)	595 (44.9)	569 (41.5)	526 (43.5)	502 (44.0)
Former	403 (35.7)	475 (39.6)	513 (36.8)	396 (33.4)	474 (39.1)	528 (39.9)	486 (35.5)	455 (37.7)	465 (40.7)
Smoker	295 (26.1)	204 (17.0)	206 (14.8)	286 (24.2)	214 (17.7)	202 (15.3)	315 (23.0)	227 (18.8)	174 (15.3)
Alcohol intake (units/week), median (IQR)	6 (2–13)	3 (1–8)	2 (0–6)	4 (1–8)	4 (1–9)	3 (0–7)	5 (1–10)	3 (0–8)	3 (0–7)
Alcohol intake (categories), *n* (%)									
Non-drinker	194 (17.2)	283 (23.6)	408 (29.2)	295 (24.9)	247 (20.4)	332 (25.1)	253 (18.4)	307 (25.4)	308 (26.9)
Low	389 (34.5)	523 (43.5)	686 (49.2)	493 (41.7)	519 (42.8)	568 (42.9)	559 (40.8)	515 (42.6)	495 (43.4)
Moderate	271 (24.0)	245 (20.4)	231 (16.5)	257 (21.7)	251 (20.7)	255 (19.2)	327 (23.9)	230 (19.0)	210 (18.4)
High	274 (24.3)	149 (12.5)	69 (5.0)	139 (11.8)	196 (16.1)	170 (12.8)	232 (16.9)	157 (13.0)	128 (11.3)
Physical activity (total minutes/day), mean (SD)	440.5 (175.2)	439.2 (155.4)	455.3 (145.0)	444.7 (174.8)	439.6 (154.5)	447.2 (145.1)	433.6 (165.7)	447.4 (155.8)	453.2 (153.0)
BMI, kg/m^2^, mean (SD)	26.5 (4.3)	26.0 (4.7)	25.2 (4.5)	26.5 (4.5)	26.0 (4.5)	25.2 (4.4)	26.3 (4.3)	25.8 (4.7)	25.4 (4.5)
Family history of CVD, *n* (%)	349 (31.0)	476 (39.7)	650 (46.6)	384 (32.4)	456 (37.7)	621 (46.8)	447 (32.6)	493 (40.8)	491 (43.0)
Family history of diabetes, *n* (%)	389 (34.5)	405 (33.7)	460 (33.0)	403 (34.0)	420 (34.6)	429 (32.4)	458 (33.4)	423 (35.0)	363 (31.8)
Type 2 diabetes, *n* (%)	109 (9.7)	106 (8.8)	111 (8.0)	103 (8.7)	120 (9.9)	117 (8.8)	113 (8.2)	121 (10.0)	99 (8.7)
Hypertension, *n* (%)	434 (38.5)	464 (38.7)	550 (39.5)	416 (35.1)	497 (41.0)	550 (41.5)	502 (36.6)	491 (40.7)	431 (37.7)
Hypercholesterolemia, *n* (%)	805 (71.5)	788 (65.6)	943 (67.7)	850 (71.8)	790 (65.2)	899 (67.8)	969 (70.7)	802 (66.4)	763 (66.9)
Dieting, *n* (%)	253 (22.4)	389 (32.4)	537 (38.5)	278 (23.5)	385 (31.8)	523 (39.4)	329 (24.0)	392 (32.5)	439 (38.4)
Dietary supplements use, *n* (%)	24 (2.2)	96 (8.0)	97 (6.9)	31 (2.6)	86 (7.1)	96 (7.2)	46 (3.4)	78 (6.5)	81 (7.1)

Abbreviations: IQR, Iinterquartile range. S.D., standard deviation. B.M.I., body mass index. kg, kilogram. m^2^, square meter. mm Hg, millimeter of mercury. CVD, cardiovascular disease. Baseline characteristics (except age and sex) were adjusted for age and sex using inverse probability weighting. Values are expressed as mean (standard deviation), median (interquartile range) for continuous variables, or number of participants (percentage) for categorical variables.

**Table 2:** Hazard ratios (95% confidence interval) for the association between tertiles of the hDPI, the DPI, and the hPBD and incident cardiovascular disease (CVD). The CoLaus Study, Lausanne, Switzerland, 2009–2021.

	Tertile 1	Tertile 2	Tertile 3	
		H.R. (95% CI)	H.R. (95% CI)	*p*-Trend
**Healthy Dietary Phytochemical Index (hDPI)**				
Incident CVD (*N* cases)	101	72	90	
Person-years	9779	10,186	10,251	
Incidence rate * (CI)	10.2 (8.4, 12.4)	7.0 (5.6, 8.9)	8.7 (7.1, 10.7)	
Crude model	1.00 (reference)	0.64 * (0.47, 0.86)	0.64 * (0.48, 0.85)	0.005
Model 1	1.00 (reference)	0.71 * (0.52, 0.96)	0.82 (0.61, 1.11)	0.275
Model 2	1.00 (reference)	0.72 * (0.53, 0.99)	0.89 (0.66, 1.21)	0.570
Model 3	1.00 (reference)	0.70 * (0.51, 0.95)	0.84 (0.62, 1.14)	0.362
**Dietary Phytochemical Index (DPI)**				
Incident CVD (*N* cases)	96	80	87	
Person-years	10,113	10,053	10,050	
Incidence rate * (CI)	9.4 (7.7, 11.5)	7.8 (6.3, 9.7)	8.6 (7, 10.6)	
Crude model	1.00 (reference)	0.73 * (0.54, 0.98)	0.67 * (0.50, 0.90)	0.011
Model 1	1.00 (reference)	0.77 (0.57, 1.05)	0.79 (0.59, 1.07)	0.153
Model 2	1.00 (reference)	0.85 (0.62, 1.15)	0.89 (0.66, 1.21)	0.514
Model 3	1.00 (reference)	0.81 (0.59, 1.10)	0.84 (0.62, 1.14)	0.303
**Healthy Plant-Based Diet Index (hPBD)**				
Incident CVD (*N* cases)	103	90	70	
Person-years	11,372	9850	8994	
Incidence rate * (CI)	8.9 (7.3, 10.8)	9.1 (7.4, 11.2)	7.7 (6.1, 9.8)	
Crude model	1.00 (reference)	0.87 (0.66, 1.16)	0.68 * (0.50, 0.93)	0.015
Model 1	1.00 (reference)	0.95 (0.71, 1.26)	0.80 (0.58, 1.09)	0.155
Model 2	1.00 (reference)	0.97 (0.72, 1.29)	0.83 (0.60, 1.15)	0.271
Model 3	1.00 (reference)	0.92 (0.69, 1.23)	0.81 (0.58, 1.12)	0.206

Abbreviations: *n*, number. CI, confidence interval. * per 1000; *p*-value: * ≤ 0.05; Model 1: adjusted by age and sex. Model 2: additionally adjusted by educational level, smoking status, alcohol consumption, physical activity, B.M.I., total caloric intake (only for the hPBD), and dieting. Model 3: additionally adjusted by type 2 diabetes, hypertension, hypercholesterolemia, and family history of CVD.

**Table 3:** Hazard ratios (95% confidence interval) for the association between tertiles of the hDPI, the DPI, and the hPBD and all-cause mortality and CVD mortality. The CoLaus Study, Lausanne, Switzerland, 2009–2021.

	All-Cause Mortality				Cardiovascular Disease Mortality			
	Tertile 1	Tertile 2	Tertile 3		Tertile 1	Tertile 2	Tertile 3	
		H.R. (95% CI)	H.R. (95% CI)	*p-*Trend		H.R. (95% CI)	H.R. (95% CI)	*p-*Trend
**Healthy Dietary Phytochemical Index (hDPI)**								
Cases	60	62	56		14	16	19	
Person-years	10,046	10,443	10,468		10,088	10,459	10,477	
Incidence rate * (CI)	5.9 (4.6, 7.6)	5.9 (4.6, 7.6)	5.3 (4.1, 6.9)		1.4 (0.8, 2.3)	1.5 (0.9, 2.4)	1.8 (1.1, 2.8)	
Crude model	1.00 (reference)	0.87 (0.61, 1.25)	0.68 * (0.47, 0.98)	0.034	1.00 (reference)	0.94 (0.45, 1.96)	0.89 (0.44, 1.78)	0.738
Model 1	1.00 (reference)	0.95 (0.66, 1.36)	0.79 (0.54, 1.15)	0.206	1.00 (reference)	1.09 (0.52, 2.27)	1.15 (0.57, 2.36)	0.699
Model 2	1.00 (reference)	0.96 (0.66, 1.39)	0.88 (0.60, 1.30)	0.509	1.00 (reference)	1.05 (0.49, 2.21)	1.31 (0.61, 2.78)	0.465
Model 3	1.00 (reference)	0.93 (0.64, 1.34)	0.84 (0.56, 1.24)	0.367	1.00 (reference)	1.06 (0.50, 2.24)	1.25 (0.59, 2.65)	0.546
**Dietary Phytochemical Index (DPI)**								
Cases	55	65	58		12	21	16	
Person-years	10,394	10,303	10,261		10,412	10,342	10,270	
Incidence rate * (CI)	5.3 (4.0, 6.8)	6.3 (4.9, 8.0)	5.6 (4.3, 7,3)		1.1 (0.6, 2.0)	2.0 (1.3, 3.1)	1.5 (0.9, 2.5)	
Crude model	1.00 (reference)	1.17 (0.81, 1.68)	0.90 (0.61, 1.31)	0.461	1.00 (reference)	1.71 (0.82, 3.58)	1.04 (0.48, 2.25)	0.832
Model 1	1.00 (reference)	1.17 (0.81, 1.68)	0.96 (0.66, 1.41)	0.755	1.00 (reference)	1.79 (0.86, 3.73)	1.19 (0.55, 2.58)	0.858
Model 2	1.00 (reference)	1.29 (0.88, 1.88)	1.11 (0.75, 1.65)	0.712	1.00 (reference)	2.12 (0.99, 4.54)	1.55 (0.68, 3.55)	0.415
Model 3	1.00 (reference)	1.27 (0.87, 1.86)	1.07 (0.72, 1.59)	0.862	1.00 (reference)	2.21* (1.01, 4.80)	1.49 (0.65, 3.43)	0.518
**Healthy Plant-Based Diet Index (hPBD)**								
Cases	67	64	47		16	17	16	
Person-years	11,683	10,120	9155		11,705	10,137	9182	
Incidence rate * (CI)	5.7 (4.5, 7.2)	6.3 (4.9, 8.0)	5.1 (3.8, 6.8)		1.3 (0.8, 2.2)	1.6 (1.0, 2.6)	1.7 (1.0, 2.8)	
Crude model	1.00 (reference)	0.98 (0.69, 1.38)	0.70 (0.48, 1.02)	0.064	1.00 (reference)	1.01 (0.51, 2.01)	0.87 (0.42, 1.79)	0.694
Model 1	1.00 (reference)	0.99 (0.70, 1.40)	0.75 (0.51, 1.10)	0.142	1.00 (reference)	1.08 (0.54, 2.15)	1.03 (0.50, 2.13)	0.949
Model 2	1.00 (reference)	1.02 (0.72, 1.45)	0.81 (0.54, 1.22)	0.323	1.00 (reference)	1.12 (0.56, 2.26)	1.03 (0.47, 2.26)	0.934
Model 3	1.00 (reference)	0.96 (0.67, 1.36)	0.77 (0.51, 1.16)	0.209	1.00 (reference)	1.14 (0.56, 2.30)	0.93 (0.42, 2.04)	0.843

* per 1000; *p*-value: * ≤0.05; Model 1: adjusted by age and sex. Model 2: additionally adjusted by educational level, smoking status, alcohol consumption, physical activity, B.M.I., total caloric intake (only for the hPBD), and dieting. Model 3: additionally adjusted by type 2 diabetes, hypertension, hypercholesterolemia, family history of CVD, and family history of diabetes.

## Data Availability

The data of CoLaus|PsyCoLaus study used in this article cannot be fully shared as they contain potentially sensitive personal information on participants. According to the Ethics Committee for Research of the Canton of Vaud, sharing these data would be a violation of the Swiss legislation with respect to privacy protection. However, coded individual-level data that do not allow researchers to identify participants are available upon request to researchers who meet the criteria for data sharing of the CoLaus|PsyCoLaus Datacenter (C.H.U.V., Lausanne, Switzerland). Any researcher affiliated to a public or private research institution who complies with the CoLaus|PsyCoLaus standards can submit a research application to research.colaus@chuv.ch or research.psycolaus@chuv.ch. Proposals requiring baseline data only, will be evaluated by the baseline (local) Scientific Committee (S.C.) of the CoLaus and PsyCoLaus studies. Proposals requiring follow-up data will be evaluated by the follow-up (multicentric) S.C. of the CoLaus|PsyCoLaus cohort study. Detailed instructions for gaining access to the CoLaus|PsyCoLaus data used in this study are available at www.colaus-psycolaus.ch/professionals/how-to-collaborate/.

## Supplementary Materials

The following supporting information can be downloaded at: https://www.mdpi.com/article/10.3390/nu15224793/s1, Annex S1. Clinical data collection in the CoLaus Study; Figure S1. Spline curves for multivariable associations of the hDPI, the DPI, and the hPBD with incident CVD. CoLaus study, Lausanne, Switzerland, 2009–2012; Figure S2A. Adjusted Nelson–Aelen cumulative risk curves for all-cause mortality. CoLaus study, Lausanne, Switzerland, 2009–2021; Figure S2B. Adjusted Nelson–Aelen cumulative risk curves for CVD mortality. CoLaus study, Lausanne, Switzerland, 2009–2021; Figure S3A. Spline curves for multivariable associations of the hDPI, the DPI, and the hPBD with all-cause mortality. CoLaus study, Lausanne, Switzerland, 2009–2012; Figure S3B. Spline curves for multivariable associations of the hDPI, the DPI, and the hPBD with CVD mortality. CoLaus study, Lausanne, Switzerland, 2009–2012; Table S1. Food items constituting the DPI and the hPBD. CoLaus study, Lausanne, Switzerland, 2009–2012; Table S2. Baseline characteristics of included and excluded participants. CoLaus study, Lausanne, Switzerland, 2009–2012; Table S3. Baseline frequency intake of food groups across tertiles of the hDPI, the DPI, and the hPBD. The CoLaus study, Lausanne, Switzerland, 2009–2012. Table S4. Hazard ratios (95% confidence interval) for the stratified analyses of multivariable associations between tertiles of the hDPI, the DPI, and the hPBD and incident cardiovascular disease (CVD). CoLaus study, Lausanne, Switzerland, 2009–2021; Table S5. Hazard ratios (95% confidence interval) for the stratified analyses of multivariable associations between tertiles of the hDPI, the DPI, and the hPBD and all-cause mortality. CoLaus study, Lausanne, Switzerland, 2009–2021; Table S6. Subhazard ratios (95% confidence interval) for the competing risk analyses of associations between tertiles of the hDPI, the DPI, and the hPBD and CVD mortality. CoLaus study, Lausanne, Switzerland, 2009–2021. References [49,50,51,52] are cited in the supplementary materials.
