# Supplementary material for: Association between Total Dietary Phytochemical Intake and Cardiometabolic Health Outcomes—Results from a 10-Year Follow-Up on a Middle-Aged Cohort Population
Source: Nutrients. 2023 Nov 15;15(22):4793. doi: 10.3390/nu15224793 (PMC10674839; doi:10.3390/nu15224793)
Supplement: Supplementary file 1 [file nutrients-15-04793-s001.zip › nutrients-2693256-supplementary.pdf]

Online Supporting Information

Association between Total Dietary Phytochemical Intake and Cardiometabolic Health Outcomes—Results from a 10-Year Follow-Up on a Middle-Aged Cohort Population

Magda Gamba [1,2,*], Octavio Pano [1,3], Peter Francis Raguindin [4], Zayne M. Roa-Diaz [5,6], Taulant Muka [1,7], Marija Glisic [1,8], Oscar H. Franco [1,9] and Pedro Marques-Vidal [10]

[1] Institute of Social and Preventive Medicine (I.S.P.M.), University of Bern, 3012 Bern, Switzerland
[2] Graduate School for Health Sciences, University of Bern, 3012 Bern, Switzerland
[3] Navarra Institute for Health Research (IdiSNA), 31009 Pamplona, Spain
[4] Faculty of Health Sciences and Medicine, University of Lucerne, 6005 Lucerne, Switzerland
[5] Medical Library, University of Bern, 3012 Bern, Switzerland
[6] Instituto Proinapsa, Universidad Industrial de Santander, Bucaramanga 680002, Colombia
[7] Epistudia, 3008 Bern, Switzerland
[8] Swiss Paraplegic Research, 6207 Nottwil, Switzerland
[9] Julius Center for Health Sciences and Primary Care, University Medical Center Utrecht, 3508 GA Utrecht, The Netherlands
[10] Department of Medicine, Internal Medicine, Lausanne University Hospital (C.H.U.V.) and University of Lausanne, 1011 Lausanne, Switzerland
* Correspondence: magda.gamba@yahoo.com

Annex S1. Clinical data collection in the CoLaus Study

From : Beuret H, Hausler N, Nanchen D, Méan M, Marques-Vidal P, Vaucher J. Comparison of Swiss and European risk algorithms for cardiovascular prevention in Switzerland. Eur J Prev Cardiol. 2021;28(2):204–10. doi: 10.1177/2047487320906305

Participants from CoLaus study were invited to attend the outpatient clinic at the University Hospital of Lausanne (CHUV, Lausanne, Switzerland) in the morning after overnight fasting for clinical assessment and questionnaires completion.

Prevalent and incident CV events were recorded through a stepwise process.

- First, relevant medical records of participants who declared, during the baseline and/or follow-up examinations, to have presented a CVD and/or CVD-related procedure during their lifetime, including MI, angina pectoris, stroke, arrhythmia, cardiomyopathy, coronarography and/or percutaneous transluminal coronary angioplasty (PTCA) and/or coronary stenting, coronary artery bypass grafting (CABG) and peripheral artery disease (PAD), were collected. The records were collected from general practitioners, cardiologists, neurologists and/or hospitals (as appropriate), and encompassed medical and/or surgical notes, laboratory, radiological, echocardiographic and electrocardiographic reports. If necessary, the original coronarography (angiogram) and brain CT/MRI exams were collected.
- Second, to retrieve events that may not have been mentioned during interviews, the central medical database of the University Hospital of Lausanne, which is the main community hospital in the catchment area of the study, was searched. Participants with hospital records were identified by cross-checking with administrative data and events of interest were detected using the following ICD-10 (*International Classification of diseases, Tenth Edition*) codes: I20.0, I21.-, I22.-, I24.-, I25.1-, I25.2-, I25.5, I25.6, I25.8, I25.9, I61.-, I62.-, I63.-, I64, I69.1, I69.2, I69.3, I69.4, I69.8, and G45.-.
- Third, death was established using the population register of the city where the participant was living in case of returned mail, absence of response when calling and/or indication from a relative. Information on cause of death was sequentially collected from: 1) general practitioners; 2) medical database of the hospital where the death occurred (either in Switzerland or abroad); 3) database of the pre-hospital emergency care unit of the City of Lausanne; 4) database of the University Centres of Forensic Medicine of Lausanne and Geneva; 5) official death certificates from the Swiss federal office of statistics; 6) verbal autopsy with a relative of the dead participant, if all previous steps failed.

Adjudication of CV events

Coronary events

All coronary-related events were adjudicated by two cardiologists based upon an international expert consensus document.[1] For this study, we defined major coronary events as a composite outcome including: i) acute coronary syndromes (ACS) (acute myocardial infarction (AMI) or unstable angina) and ii) symptomatic stable angina followed by a revascularization procedure, either by percutaneous coronary intervention (PCI) or by coronary artery bypass grafting (CABG). Iatrogenic events caused by a medical procedure (angioplasty, stenting or coronary artery bypass surgery) were not considered. The following criteria were applied:

A. Definite AMI, including ST-segment elevation myocardial infarction (STEMI) and Non-ST-segment elevation myocardial infarction and (NSTEMI) was defined in the presence of at least one of the following criteria:

   1. Detection of rise/fall of Troponin I or T with at least one value above the 99th percentile of the upper reference limit together with evidence of myocardial ischaemia with *at least one* of the following:
      - Symptoms of ischaemia
      - ECG changes indicative of new ischaemia (new ST-T changes or new left bundle branch block)
      - Development of pathological Q waves in the ECG
      - Echocardiographic evidence of new segmental abnormality

   2. Sudden, unexpected cardiac death, involving cardiac arrest, often with symptoms suggestive of myocardial ischaemia, and accompanied by presumably new ST elevation, or new left bundle branch block

      and/or

      - Evidence of fresh thrombus by coronary angiography and/or at autopsy

      but

      - Occurring before blood samples could be obtained, or at a time before the appearance of cardiac biomarkers in the blood (Troponin I or T).

B. Due to missing information on cardiac biomarkers, probable AMI was defined using the following criteria:

   1. Symptoms of ischaemia

      and

   2. ECG changes: new ST-T changes, new left bundle branch block or new pathological Q waves

      and/or

3. Coronary angiography followed by percutaneous or surgical revascularization

   and/or

4. Echocardiographic evidence of new segmental abnormality

C. Unstable angina was defined using the following criteria (used by Cardiobase/SPUM)**:**

1. Symptoms of ischaemia
2. Hospitalization or ambulatory care
3. Negative troponin values
4. Change in medication after hospital discharge or during ambulatory care (betablocker, calcium blocker, nitro-derivatives, molsidomine, nicorandil)

D. Coronary artery disease (CAD) events corresponded to participants who presented with typical symptoms (stable angina) and underwent either percutaneous (PTCA ± stenting) or surgical (CABG) revascularizations, unless these procedures were directly related to an AMI. When the exact date of the event could not be traced, the date of the revascularization was used (n=4). The following criteria were used to define CAD:

1. Symptoms of ischaemia

and

2. Coronary angiography followed by percutaneous or surgical revascularization

without:

- Acute context
- ECG changes compatible with AMI
- Modification in medication (-> unstable angina).

3. Detection of rise/fall of troponin I or T

Strokes and transient ischemic attacks

Fatal and nonfatal strokes were adjudicated by one neurologist in participants who presented non-traumatic and rapidly progressing focal or global disturbances of cerebral function lasting ≥24h.[2, 3]

Ischaemic origin was based upon normal imaging or imaging (CT and/or MRI) showing a recent lesion of ischaemic nature and compatible with the clinical presentation. Ischaemic strokes with haemorrhagic conversion were also listed here. Haemorrhagic origin was based upon imaging (CT and/or MRI) showing the presence of intracerebral, intraventricular and/or subarachnoid blood of presumed spontaneous occurrence and compatible with the clinical presentation. Transient ischaemic attacks were defined upon rapidly developing focal or global disturbances of cerebral function of presumed vascular origin and lasting <24 hours.[4]

Stroke was defined according to WHO definition [3], i.e. as rapidly developing clinical signs of focal or global disturbance of cerebral function of presumed vascular origin lasting ≥ 24 hours

- *Ischemic* : normal imaging or imaging showing recent lesion of ischemic nature correponding to clinical syndrome. Ischemic strokes with conversion to haemorrhage are listed here.
- *Haemorrhagic* : intracerebral and/or intraventricular and/or subarachnoid blood of presumed spontaneous appearance and corresponding to clinical syndrome.

- Transient ischemic attack [4] was defined as rapidly developing clinical signs of focal or global disturbance of presumed vascular origin lasting < 24 hours.
  - *With normal imaging*
  - *With imaging showing recent lesion* of presumed ischemic nature and correponding to clinical syndrome.

Remarks:

- Not due to traumatism and not due to transformation of an ischemic stroke. Non-traumatic symptomatic subarachnoid haemorrhagic is considered a haemorrhagic stroke.
- Traditionally, the TIA definition was purely clinical (complete disappearance of symptoms within 24 hours). In a new proposal [4], a TIA with pathological imaging is considered a « stroke », and a TIA without pathological imaging is considered a « TIA » (= a transient episode of neurological dysfunction caused by focal brain, spinal cord, or retinal ischemia, without acute infarction). The problem with this new definition is that it depends strongly on the type of imaging used.
- An ophthalmologist's exam related to the event is mandatory to exclude other diseases and to confirm the arterial occlusion.

The following cerebrovascular diseases were not considered as outcome events:

1. Spinal cord ischemia
2. Retinal artery occlusion (central retinal artery or branch retinal artery) : rapidly developed clinical signs of disturbance of retinal function of presumed vascular origin lasting ≥ 24 hours.[2]
3. *Amaurosis fugax* of presumed arterial origin: rapidly developed clinical signs of disturbance of retinal function of presumed vascular origin lasting ≥ 24 hours.[2]
4. Cerebral sinus vein thrombosis.
5. Intracranial vascular malformations with other than ischemic or haemorrhagic manifestations.

Cardiovascular deaths

Deaths were adjudicated by two internists and were classified as cardiac, vascular and non-cardiovascular; deaths from cerebrovascular origin were already defined as previously mentioned. Cardiac deaths, including fatal MI and cardiac sudden deaths, were based upon the same criteria as non-

fatal MI.[1] Vascular deaths encompassed aortic dissection, valvular heart disease, fatal arrhythmia and cardiac failure. Non-cardiovascular deaths included all other diagnoses not listed above (e.g. accident, infection/sepsis, cancer, pulmonary embolism, suicide, etc.). Undetermined deaths were defined as deaths that occurred outside hospital with or without witness. Unless another diagnosis could be established, they were listed as cardiac if their origin could be reasonably attributable to a coronary event, for example by the presence of typical symptoms just before death and/or personal history (presence of ≥2 traditional CV risk factors and/or ACS or CAD).

References

49. Thygesen, K.; Alpert, J.S.; White, H.D. *Universal definition of myocardial infarction.* Eur Heart J, 2007. **28**(20): p. 2525-38.

50. Advisory Council for the National Institute of Neurological and Communicative Disorders and Stroke. National Institute of Health. *A classification and outline of cerebrovascular diseases. II.* Stroke, 1975. **6**(5): p. 564-616.

51. World Health Organization, *Cerebrovascular disorders: a clinical and research classification.WHO offset publication 1978; no. 43. www.who.int/iris/handle/10665/37194#sthash.I8sxekCh.dpuf (Accessed August 19, 2015).* 1978.

52. Easton, J.D.; Saver, J.L.; Albers, G.W.; Alberts, M.J.; Chaturvedi, S.; Feldmann, E.; Hatsukami, T.S.; Higashida, R.T.; Johnston, S.C.; Kidwell, C.S. et al. *Definition and evaluation of transient ischemic attack: a scientific statement for healthcare professionals from the American Heart Association/American Stroke Association Stroke Council; Council on Cardiovascular Surgery and Anesthesia; Council on Cardiovascular Radiology and Intervention; Council on Cardiovascular Nursing; and the Interdisciplinary Council on Peripheral Vascular Disease. The American Academy of Neurology affirms the value of this statement as an educational tool for neurologists.* Stroke, 2009. **40**(6): p. 2276-93.

Table S1. Food items constituting DPI and hPBD. CoLaus study, Lausanne, Switzerland, 2009–2012.

Group and food items	
Dietary Phytochemical Index (DPI)	
Whole grains	Whole wheat bread, rye bread, and muesli
Vegetables	Green beans, spinach, cauliflower, broccoli, tomatoes, carrots, green salad, green peas, corn, maize, avocado, natural vegetable soups, tomato sauce, and tofu
Fruits	Banana, apple, pear, plum, grapes, orange, mandarine, peach, apricot, melon, berries, kiwi, preserved fruit, and fresh fruit juice
Olive oil (for cooking)	
*Alcohol**	Beer, wine, and champagne
Healthy Plant-Based Diet Index (hPBD)	
Healthy food groups	
Whole grains	Whole wheat bread, rye bread, and muesli
Vegetables	Green beans, spinach, cauliflower, broccoli, tomatoes, carrots, green salad, green peas, corn, maize, avocado, natural vegetable soups, tomato sauce, and tofu
Fruits	Banana, apple, pear, plum, grapes, orange, mandarine, peach, apricot, melon, berries, kiwi, preserved fruit, and fresh fruit juice
Vegetable oils	Olive oil (for cooking), peanut oil (for cooking) and sunflowerseed oil (for cooking)
Tea & Coffee	Tea, herbal tea and coffee
Less healthy	
Bottled Fruit Juice	Bottled Fruit Juice
Refined grains	White or soft bread, tresse, corn-flakes, puffed rice, rusks, Swedish bread, white rice, pasta, ravioli, tortellini, cannelloni, couscous, semolina, croissant, and chocolate bread
Potatoes	Potatoes (boiled) and french fries
Sugar sweetened beverages	Lemonade, soda and syrup
Sweets and Desserts	Fruit tart, cream tart, cream cake, cake, dried pastries, biscuits, cookies, chocolate, honey, and jam
Animal food groups	
Animal fat	Butter (spreading and for cooking)
Dairy	Plain yogurth, low-fat yogurth, fruit/aroma yogurth, cottage cheese (0% fat), cottage cheese (regular fat), ricotta, cheese fondue, feta, mozzarella, gruyere, tomme, camembert, cream (35% fat), ice cream, sorbet and milk as drink (regular and 0% fat)
Egg	Eggs
Fish or seafood	Salmon (fresh or smoked), fried or breaded fish, tuna in oil, White fish (cod, trout, hake), and seafood (shrimps, mussels)
Meat	Beef, horse, veal, chicken (with skin and skinned), beef horse or veal (hamburger, rib steak, and roasted meat), cured ham, mutton or pork chops, sausage, salami, ham, cervelas, wieners, pâté, terrine, frankfurter, small sausages, liver (veal, pork or poultry) and offalls
Miscellaneous animal-based foods	Pizza and mayonnaise sauce

* Healthy DPI (hDPI) is constituted as DPI but excludes alcohol items.

Table S2. Baseline characteristics of included and excluded participants. CoLaus study, Lausanne, Switzerland, 2009-2012.

	Excluded (n = 1343)	*Included (n = 3721)*	*p Value*
Healthy Dietary Phytochemical Index (%), mean (SD)	22.2 (13.1)	23.4 (12.5)	0.010
Dietary Phytochemical Index (%), mean (SD)	26.4 (13.2)	26.9 (12.3)	0.335
Healthy Plant-Based Diet Index, mean (SD)	46.9 (6.8)	48.3 (7.0)	< 0.001
Age, mean (SD)	59.2 (10.8)	57.2 (10.4)	< 0.001
Sex, n (% women)	667 (49.7%)	2040 (54.8%)	0.001
Education attainment, n (%)			< 0.001
University education	219 (16.3%)	860 (23.1%)	
High school	290 (21.6%)	1016 (27.3%)	
Apprenticeship	486 (36.2%)	1310 (35.2%)	
Mandatory education	348 (25.9%)	535 (14.4%)	
Smoking status, n (%)			< 0.001
Never	464 (34.5%)	1572 (42.2%)	
Former	471 (35.1%)	1413 (38.0%)	
Smoker	408 (30.4%)	736 (19.8%)	
Alcohol intake (units/week), mean (SD)	6.3 (9.2)	6.4 (8.0)	0.613
Physical activity (total minutes/day), mean (SD)	440.1 (142.9)	443.5 (161.2)	0.492
BMI, kg/m2, mean (SD)	26.9 (4.8)	26.0 (4.5)	< 0.001
Family history of cardiovascular disease, n (%)	662 (49.3%)	1403 (37.7%)	< 0.001
Family history of type 2 diabetes, n (%)	587 (43.7%)	1247 (33.5%)	< 0.001
Cardiovascular disease prevalence, n (%)	162 (12.1%)	184 (4.9%)	< 0.001
Type 2 diabetes prevalence, n (%)	209 (15.6%)	340 (9.1%)	< 0.001
Hypertension prevalence, n (%)	692 (51.5%)	1413 (38.0%)	< 0.001
Hypercholesterolemia prevalence, n (%)	815 (60.7%)	2523 (67.8%)	< 0.001
Special diet, n (%)	430 (32.0%)	1144 (30.7%)	0.387
Dietary supplements use, n (%)	42 (3.1%)	189 (5.1%)	0.003
Total energy intake (Kcal/day), mean (SD)	1088 (1146)	1796 (602)	< 0.001

Abbreviations: IQR, Interquartile range. BMI, Body mass index. Kg, kilogram. m2, square meter. Kcal, kilocalorie.

Values are expressed as mean ± standard deviation (SD) or as number of participants (percentage). The between-group comparisons were made using chi-square for categorical variables and t-test for continuous variables.

Table S3. Baseline frequency intake of food groups across tertiles of hDPI, DPI, and hPBD. The CoLaus study, Lausanne, Switzerland, 2009–2012.

	**Tertile 1**	**Tertile 2**	**Tertile 3**
**Healthy Dietary Phytochemical Index (hDPI)**			
Number of participants	1127	1200	1394
hDPI (%),	11 (8-14)	21 (19-24)	36 (31-43)
Total energy intake (Kcal/day),	1791 (1422-2246)	1604 (1302-2003)	1632 (1282-2024)
Total energy intake from PRFs (Kcal/day),	184 (125-260)	342 (272-440)	606 (461-784)
Whole grains (g/day),	4 (0-18)	25 (7-50)	75 (38-125)
Vegetables (g/day),	130 (90-189)	161 (116-226)	187 (128-277)
Fruits (g/day),	99 (50-178)	235 (140-359)	400 (233-590)
Olive oil (for cooking), (mL/day),	4 (1-7)	5 (2-10)	7 (4-10)
Vegetable oils intake (excluding olive oil), (mL/day),	2 (0-5)	2 (0-4)	1 (0-4)
Tea & Coffee intake (mL/day),	243 (200-400)	303 (207-500)	400 (218-750)
Bottled fruit juice (mL/day),	18 (0-100)	18 (0-100)	0 (0-43)
Refined grains (g/day),	168 (113-255)	115 (78-174)	76 (49-117)
Potatoes (g/day),	32 (17-53)	26 (13-48)	21 (13-39)
Sugar-sweetened beverages (mL/day),	18 (0-100)	7 (0-43)	0 (0-18)
Sweets & desserts (g/day),	33 (17-56)	33 (21-53)	30 (16-46)
Animal fat (g/day),	3 (0-8)	3 (0-7)	2 (0-7)
Dairy (g/day),	170 (91-277)	175 (98-271)	172 (94-269)
Eggs (g/day),	9 (4-21)	9 (4-21)	9 (4-21)
Fish & seafood (g/day),	29 (16-46)	32 (19-50)	32 (18-50)
Meat (g/day),	94 (61-128)	70 (47-101)	53 (34-77)
Miscellaneous (g/day),	15 (9-33)	12 (4-23)	9 (0-14)
Alcoholic beverages (mL/day),	86 (31-236)	47 (11-139)	32 (5-75)
**Dietary Phytochemical Index (DPI)**			
Number of participants	1184	1212	1325
DPI (%),	15 (12-18)	26 (23-28)	39 (35-46)
Total energy intake (Kcal/day),	1759 (1412-2227)	1634 (1308-2052)	1647 (1299-2035)
Total energy intake from PRFs (Kcal/day),	204 (137-281)	354 (274-464)	605 (452-792)
Whole grains (g/day),	5 (0-19)	29 (7-54)	75 (38-125)
Vegetables (g/day),	136 (92-196)	161 (113-230)	181 (124-271)
Fruits (g/day),	121 (60-203)	237 (128-376)	390 (213-587)
Olive oil (for cooking), (mL/day),	4 (1-7)	5 (2-10)	7 (4-10)
Vegetable oils intake (excluding olive oil), (mL/day),	2 (0-5)	2 (0-4)	0 (0-4)
Tea & Coffee intake (mL/day),	280 (200-400)	300 (200-500)	350 (207-700)
Bottled fruit juice (mL/day),	18 (0-100)	7 (0-100)	0 (0-43)
Refined grains (g/day),	168 (111-254)	117 (77-174)	77 (49-117)
Potatoes (g/day),	29 (16-50)	26 (13-50)	24 (13-43)
Sugar-sweetened beverages (mL/day),	18 (0-100)	0 (0-43)	0 (0-11)
Sweets & desserts (g/day),	35 (19-56)	34 (21-53)	28 (15-46)
Animal fat (g/day),	3 (0-8)	3 (0-8)	2 (0-7)

Dairy (g/day),	182 (101-295)	171 (95-268)	169 (90-263)
Eggs (g/day),	9 (4-21)	9 (4-21)	9 (4-21)
Fish & seafood (g/day),	29 (16-47)	32 (19-50)	31 (18-50)
Meat (g/day),	90 (57-126)	72 (48-101)	53 (33-78)
Miscellaneous (g/day),	14 (9-33)	13 (5-23)	9 (0-14)
Alcoholic beverages (mL/day),	46 (13-113)	48 (13-150)	43 (13-150)
**Healthy Plant-Based Diet Index (hPBD)**			
Number of participants	1371	1209	1141
hPBD, score,	42 (39-44)	48 (47-50)	56 (53-59)
Total energy intake (Kcal/day),	1626 (1277-2029)	1592 (1290-2022)	1871 (1526-2223)
Healthy group foods			
Whole grains (g/day),	11 (0-30)	32 (7-71)	66 (25-125)
Vegetables (g/day),	127 (91-177)	163 (114-230)	215 (149-303)
Fruits (g/day),	131 (65-230)	219 (115-374)	403 (237-605)
Olive oil (for cooking), (mL/day),	4 (2-7)	5 (2-10)	7 (4-10)
Vegetable oils intake, (mL/day),	5 (3-10)	7 (5-11)	10 (7-15)
Tea & Coffee intake (mL/day),	230 (180-343)	300 (200-500)	400 (280-750)
Less healthy food groups			
Bottled fruit juice (mL/day),	18 (0-100)	7 (0-64)	0 (0-43)
Refined grains (g/day),	139 (94-212)	110 (66-183)	93 (58-149)
Potatoes (g/day),	32 (18-53)	26 (13-47)	21 (11-39)
Sugar-sweetened beverages (mL/day),	18 (0-100)	0 (0-27)	0 (0-7)
Sweets & desserts (g/day),	33 (20-53)	31 (18-51)	30 (16-51)
Animal food groups			
Animal fat (g/day),	4 (1-9)	2 (0-7)	1 (0-6)
Dairy (g/day),	165 (96-261)	171 (93-266)	198 (97-303)
Eggs (g/day),	9 (5-21)	9 (4-21)	9 (4-21)
Fish & seafood (g/day),	32 (19-51)	29 (17-48)	30 (16-50)
Meat (g/day),	87 (56-118)	66 (43-98)	58 (36-89)
Miscellaneous (g/day),	16 (9-33)	10 (1-22)	9 (0-14)
Alcoholic beverages (mL/day),	64 (16-155)	43 (8-145)	32 (5-139)

Abbreviations: IQR, Interquartile range. SD, standard deviation. Kcal, kilocalorie. g, grams. mL, milliliters.
Baseline frequency intake of food groups adjusted by age and sex using inverse probability weighting.
Values are expressed as median (interquartile range)

Table S4. Hazard ratios (95% confidence interval) for the stratified analyses of multivariable associations between tertiles of hDPI, DPI, and hPBD and incident cardiovascular disease (CVD). CoLaus study, Lausanne, Switzerland, 2009–2021

	**hDPI**				**DPI**				**PBD**			
	**Tertile 1**	**Tertile 2**	**Tertile 3**	***p*-trend**	**Tertile 1**	**Tertile 2**	**Tertile 3**	***p*-trend**	**Tertile 1**	**Tertile 2**	**Tertile 3**	***p*-trend**
		HR (95% CI)				HR (95% CI)				HR (95% CI)		
**Age**												
40 - 60 years	Reference	0.77 (0.44, 1.35)	1.13 (0.63, 2.02)	0.704	Reference	1.00 (0.58, 1.72)	1.18 (0.67, 2.06)	0.569	Reference	1.15 (0.69, 1.90)	0.66 (0.35, 1.27)	0.279
> 60 years		0.67* (0.46, 0.98)	0.78 (0.54, 1.12)	0.260		0.72 (0.49, 1.04)	0.73 (0.51, 1.06)	0.132		0.89 (0.62, 1.27)	0.92 (0.63, 1.33)	0.671
**Sex**												
Women	Reference	0.65 (0.37, 1.13)	0.86 (0.51, 1.44)	0.909	Reference	0.82 (0.49, 1.39)	0.79 (0.47, 1.30)	0.374	Reference	0.80 (0.49, 1.31)	0.71 (0.43, 1.17)	0.186
Men		0.73 (0.50, 1.07)	0.79 (0.53, 1.18)	0.236		0.76 (0.52, 1.10)	0.77 (0.52, 1.14)	0.399		1.01 (0.71, 1.45)	0.84 (0.55, 1.27)	0.426
**BMI**												
Normal weight	Reference	0.61 (0.34, 1.09)	0.59 (0.34, 1.03)	0.095	Reference	0.66 (0.37, 1.17)	0.64 (0.37, 1.09)	0.130	Reference	0.76 (0.45, 1.29)	0.70 (0.40, 1.21)	0.211
Pre-obesity & obesity		0.71 (0.49, 1.03)	0.96 (0.66, 1.39)	0.902		0.85 (0.59, 1.22)	0.92 (0.63, 1.34)	0.683		0.99 (0.70, 1.40)	0.85 (0.58, 1.25)	0.423
**Family history of Diabetes**												
No history	Reference	0.68 (0.43, 1.06)	0.89 (0.57, 1.39)	0.641	Reference	0.87 (0.56, 1.35)	1.00 (0.64, 1.56)	0.987	Reference	0.78 (0.51, 1.18)	0.81 (0.52, 1.27)	0.378
History		0.73 (0.47, 1.14)	0.84 (0.54, 1.29)	0.536		0.77 (0.50, 1.20)	0.75 (0.49, 1.15)	0.224		1.04 (0.69, 1.57)	0.88 (0.56, 1.40)	0.413
**Smoking**												
Never		0.96 (0.53, 1.75)	1.25 (0.70, 2.22)	0.378		0.70 (0.39, 1.25)	1.03 (0.60, 1.76)	0.720		0.72 (0.43, 1.22)	0.74 (0.43, 1.29)	0.296
Former	Reference	0.51* (0.32, 0.82)	0.69 (0.43, 1.10)	0.175	Reference	0.71 (0.44, 1.13)	0.80 (0.50, 1.28)	0.400	Reference	1.21 (0.78, 1.88)	0.94 (0.57, 1.53)	0.774
Current		0.74 (0.40, 1.39)	0.86 (0.44, 1.69)	0.616		1.24 (0.69, 2.22)	0.67 (0.33, 1.35)	0.290		0.81 (0.45, 1.47)	0.97 (0.50, 1.88)	0.862
**Type 2 diabetes**												
No	Reference	0.62* (0.43, 0.88)	0.81 (0.58, 1.14)	0.322	Reference	0.81 (0.58, 1.15)	0.79 (0.56, 1.12)	0.205	Reference	0.91 (0.65, 1.25)	0.82 (0.58, 1.17)	0.275
Yes		0.95 (0.47, 1.91)	0.92 (0.43, 1.99)	0.841		0.74 (0.36, 1.50)	0.92 (0.44, 1.90)	0.907		0.95 (0.49, 1.84)	0.76 (0.35, 1.64)	0.476

P-value: * ≤0.05
Multivariable model adjusted by age, sex, educational level, smoking status, alcohol consumption, physical activity, BMI, total caloric intake (only for hPBD), dieting, type 2 diabetes, hypertension, hypercholesterolemia, and family history of CVD

Table S5. Hazard ratios (95% confidence interval) for the stratified analyses of multivariable associations between tertiles of hDPI, DPI, and hPBD and all-cause mortality. CoLaus study, Lausanne, Switzerland, 2009–2021

	hDPI				DPI				PBD			
	Tertile 1	Tertile 2	Tertile 3	*p*-trend	Tertile 1	Tertile 2	Tertile 3	*p*-trend	T 1	Tertile 2	Tertile 3	*p*-trend
	HR (95% CI)				HR (95% CI)				HR (95% CI)			
**Age**												
40 - 60 years	Ref	0.40 (0.13, 1.25)	1.01 (0.37, 2.72)	0.929	Ref	1.08 (0.38, 3.09)	1.79 (0.65, 4.89)	0.255	Ref	1.95 (0.72, 5.31)	1.39 (0.47, 4.09)	0.542
> 60 years		1.10 (0.73, 1.64)	0.89 (0.58, 1.36)	0.510		1.40 (0.93, 2.12)	1.08 (0.70, 1.67)	0.892		0.95 (0.65, 1.40)	0.73 (0.46, 1.15)	0.173
**Sex**												
Women	Ref	0.76 (0.40, 1.43)	0.73 (0.40, 1.32)	0.358	Ref	0.95 (0.50, 1.80)	1.10 (0.59, 2.04)	0.696	Ref	1.03 (0.59, 1.79)	0.57 (0.30, 1.11)	0.092
Men		1.05 (0.66, 1.67)	0.86 (0.50, 1.48)	0.598		1.48 (0.90, 2.42)	0.96 (0.54, 1.71)	0.891		0.97 (0.60, 1.57)	0.78 (0.45, 1.36)	0.397
**BMI**												
Normal weight	Ref	0.92 (0.44, 1.96)	0.85 (0.41, 1.79)	0.673	Ref	1.45 (0.68, 3.07)	1.20 (0.56, 2.58)	0.777	Ref	0.81 (0.42, 1.55)	0.48 (0.22, 1.05)	0.066
Pre-obesity & obesity		0.86 (0.55, 1.32)	0.74 (0.46, 1.20)	0.228		1.03 (0.65, 1.64)	0.92 (0.57, 1.49)	0.702		1.04 (0.68, 1.61)	0.90 (0.55, 1.50)	0.702
**Smoking**												
Never		1.36 (0.63, 2.97)	1.05 (0.47, 2.32)	0.888		1.71 (0.78, 3.75)	1.40 (0.61, 3.19)	0.573		0.99 (0.50, 1.96)	0.57 (0.25, 1.27)	0.160
Former	Ref	0.90 (0.53, 1.52)	0.97 (0.54, 1.72)	0.933	Ref	1.08 (0.62, 1.89)	1.11 (0.62, 1.98)	0.748	Ref	1.03 (0.61, 1.74)	0.91 (0.50, 1.64)	0.762
Current		0.57 (0.23, 1.40)	0.40 (0.16, 1.00)	0.049		1.48 (0.64, 3.40)	0.59 (0.23, 1.54)	0.306		0.87 (0.38, 1.99)	0.52 (0.19, 1.45)	0.219
**Hypertension**												
No	Ref	0.58 (0.27, 1.21)	0.75 (0.39, 1.47)	0.547	Ref	0.81 (0.39, 1.69)	1.03 (0.52, 2.01)	0.840	Ref	1.62 (0.84, 3.11)	0.73 (0.33, 1.62)	0.381
Yes		1.10 (0.71, 1.71)	0.91 (0.55, 1.48)	0.651		1.56 (0.99, 2.48)	1.12 (0.68, 1.86)	0.774		0.78 (0.51, 1.21)	0.76 (0.46, 1.27)	0.274
**Hypercholesterolemia**												
No	Ref	0.93 (0.50, 1.73)	1.06 (0.56, 2.02)	0.828	Ref	1.41 (0.75, 2.67)	1.05 (0.53, 2.07)	1.000	Ref	0.74 (0.39, 1.39)	1.06 (0.56, 2.01)	0.835
Yes		0.92 (0.57, 1.48)	0.73 (0.44, 1.22)	0.220		1.09 (0.67, 1.77)	1.08 (0.66, 1.79)	0.771		1.10 (0.70, 1.72)	0.59 (0.33, 1.05)	0.082

P-value: * ≤0.05

Multivariable model adjusted by age, sex, educational level, smoking status, alcohol consumption, physical activity, BMI, total caloric intake (only for hPBD), dieting, type 2 diabetes, hypertension, hypercholesterolemia, family history of CVD and family history of type 2 diabetes.

Table S6. Subhazard ratios (95% confidence interval) for the competing risk analyses of associations between tertiles of hDPI, DPI, and hPBD and CVD mortality. CoLaus study, Lausanne, Switzerland, 2009–2021

	**Tertile 1**	**Tertile 2** SHR (95% CI)	**Tertile 3** SHR (95% CI)
**Healthy Dietary Phytochemical Index (hDPI)**			
Crude model		1.01 (0.50, 2.04)	0.95 (0.47, 1.90)
Model 1		1.14 (0.56, 2.34)	1.21 (0.59, 2.49)
Model 2	1.00 (reference)	1.08 (0.52, 2.25)	1.29 (0.61, 2.72)
Model 3		1.11 (0.54, 2.29)	1.22 (0.59, 2.51)
**Dietary Phytochemical Index (DPI)**			
Crude model		1.52 (0.75, 3.07)	0.98 (0.46, 2.08)
Model 1		1.61 (0.79, 3.28)	1.13 (0.53, 2.42)
Model 2	1.00 (reference)	1.92 (0.91, 4.09)	1.45 (0.64, 3.26)
Model 3		2.00 (0.91, 4.36)	1.36 (0.62, 2.99)
**Healthy Plant-Based Diet Index (hPBD)**			
Crude model		1.03 (0.52, 2.05)	1.03 (0.51, 2.06)
Model 1		1.12 (0.56, 2.23)	1.22 (0.60, 2.48)
Model 2	1.00 (reference)	1.09 (0.55, 2.18)	1.15 (0.52, 2.53)
Model 3		1.12 (0.56, 2.25)	1.01 (0.47, 2.19)

Model 1: adjusted by age and sex. Model 2: additionally adjusted by educational level, smoking status, alcohol consumption, physical activity, BMI, total caloric intake (only for hPBD), and dieting. Model 3: additionally adjusted by type 2 diabetes, hypertension, hypercholesterolemia, family history of CVD, and family history of Diabetes.

Figure S1. Spline curves for multivariable associations of hDPI, DPI, and hPBD with incident CVD. CoLaus study, Lausanne, Switzerland, 2009–2012

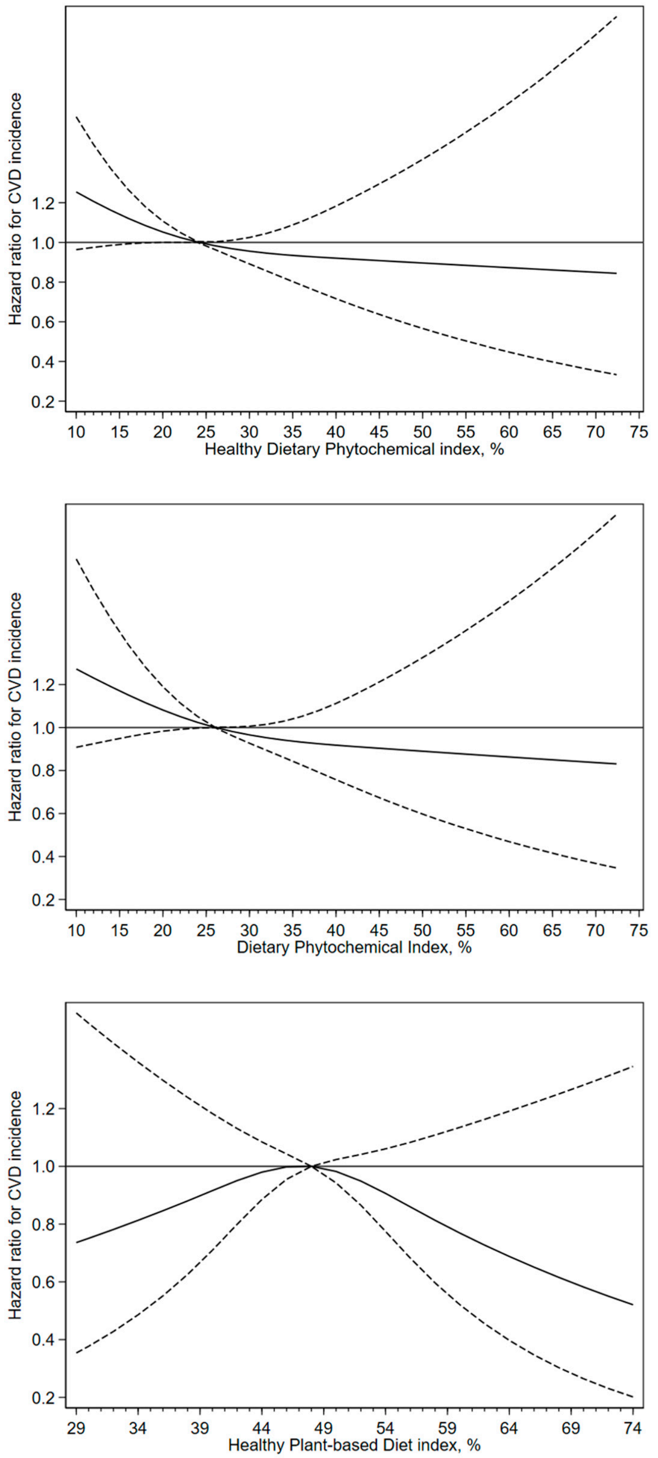

Cubic splines estimated with fully adjusted models: age, sex, educational level, smoking status, alcohol consumption, physical activity, BMI, total caloric intake (only for hPBD), dieting, T2D, hypertension, hypercholesterolemia and family history of CVD.

Figure S2A. Adjusted Nelson-Aelen cumulative risk curves for all-cause mortality. CoLaus study, Lausanne, Switzerland, 2009–2021

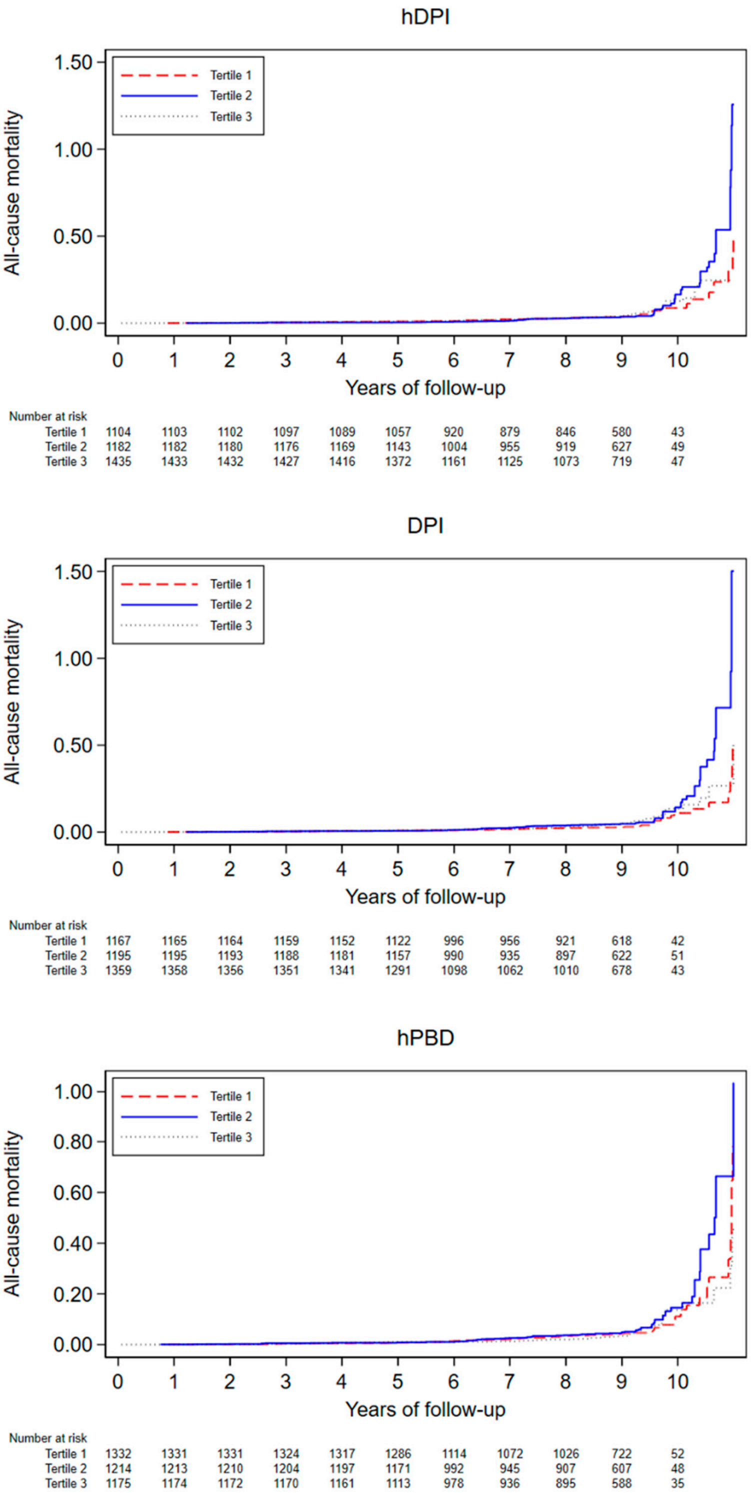

Curves are adjusted by age, sex, smoking status, physical activity, BMI, hypertension, and family history of CVD and T2D using IPW.

Figure S2B. Adjusted Nelson-Aelen cumulative risk curves for CVD mortality. CoLaus study, Lausanne, Switzerland, 2009–2021

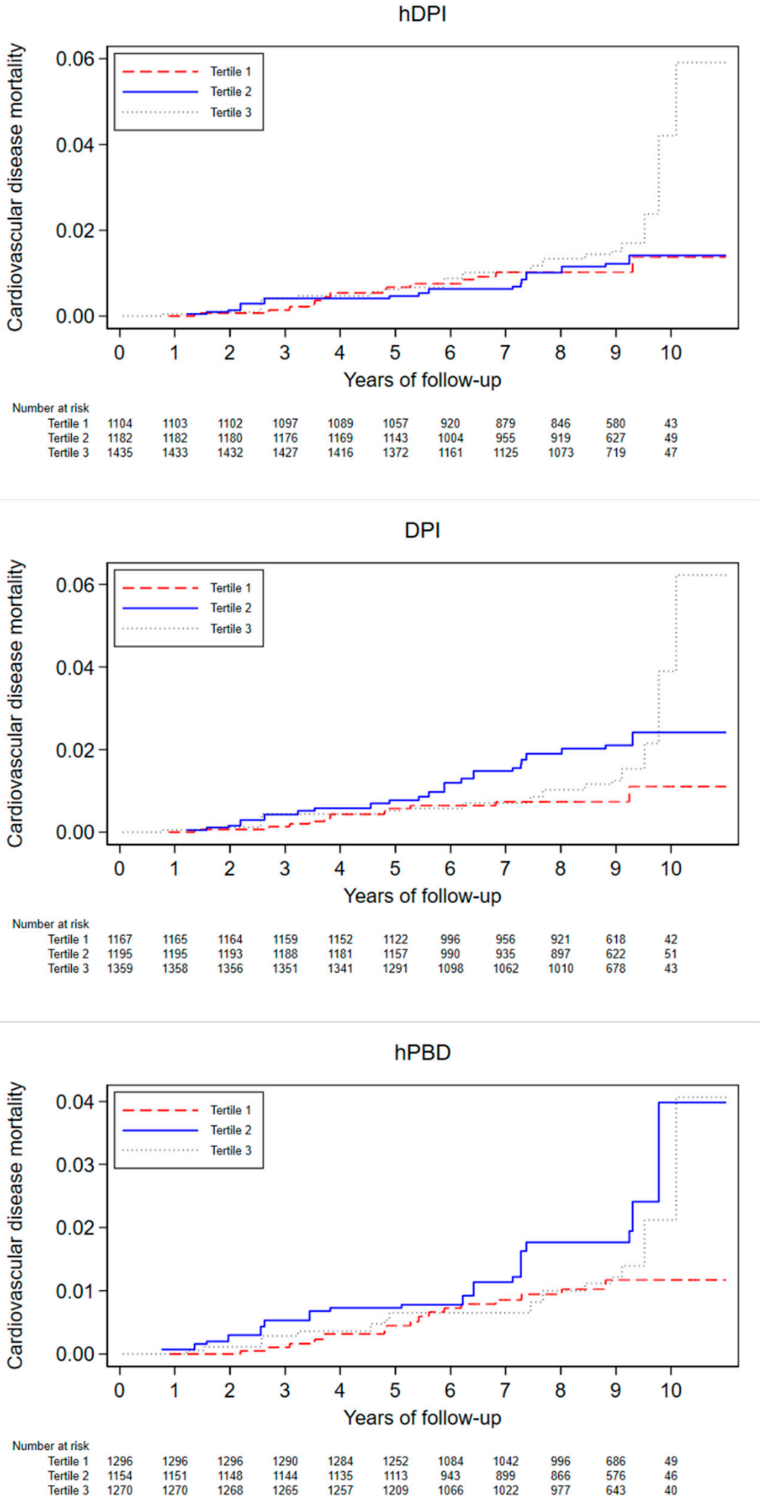

Curves are adjusted by age, sex, smoking status, physical activity, BMI, hypertension, and family history of CVD and T2D using IPW.

Figure S3A. Spline curves for multivariable associations of hDPI, DPI, and hPBD with all-cause mortality. CoLaus study, Lausanne, Switzerland, 2009–2012

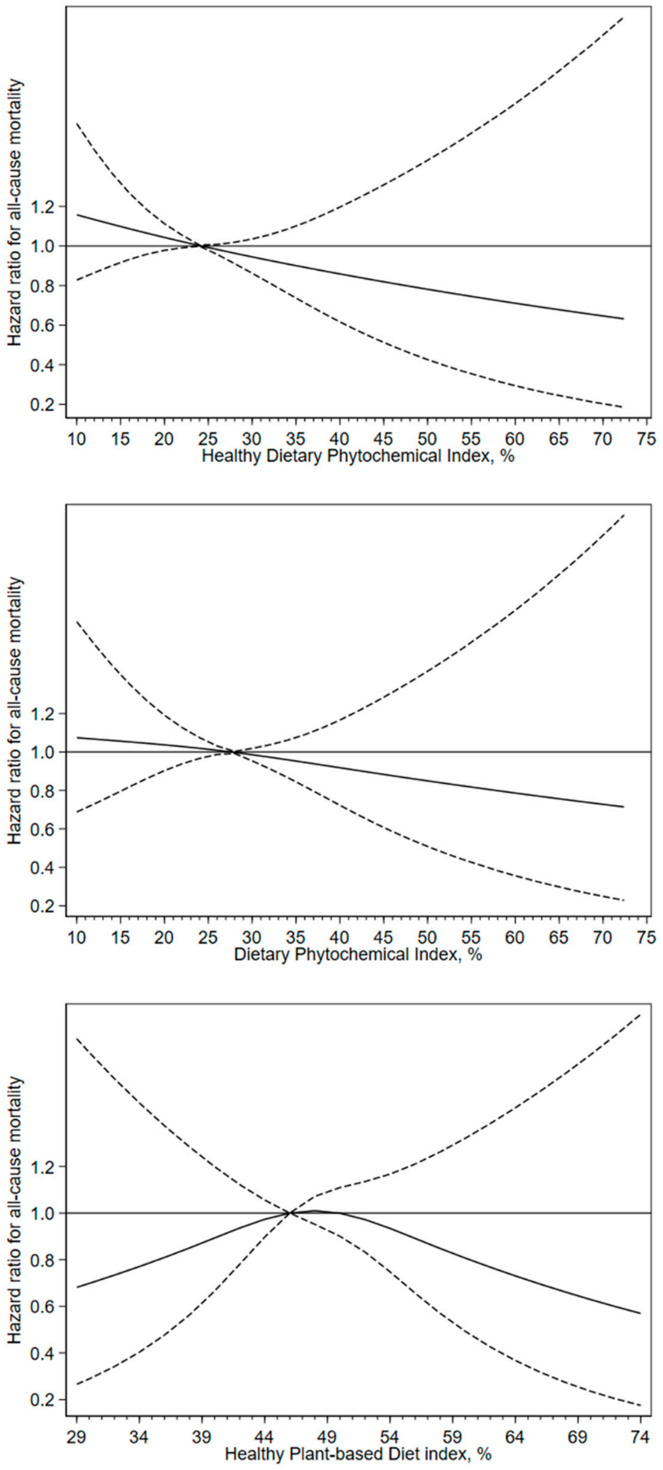

Cubic splines estimated with fully adjusted models: age, sex, educational level, smoking status, alcohol consumption, physical activity, BMI, total caloric intake (only for hPBD), dieting, type 2 diabetes, hypertension, hypercholesterolemia, family history of CVD and family history of Diabetes.

Figure S3B. Spline curves for multivariable associations of hDPI, DPI, and hPBD with CVD mortality.

CoLaus study, Lausanne, Switzerland, 2009–2012

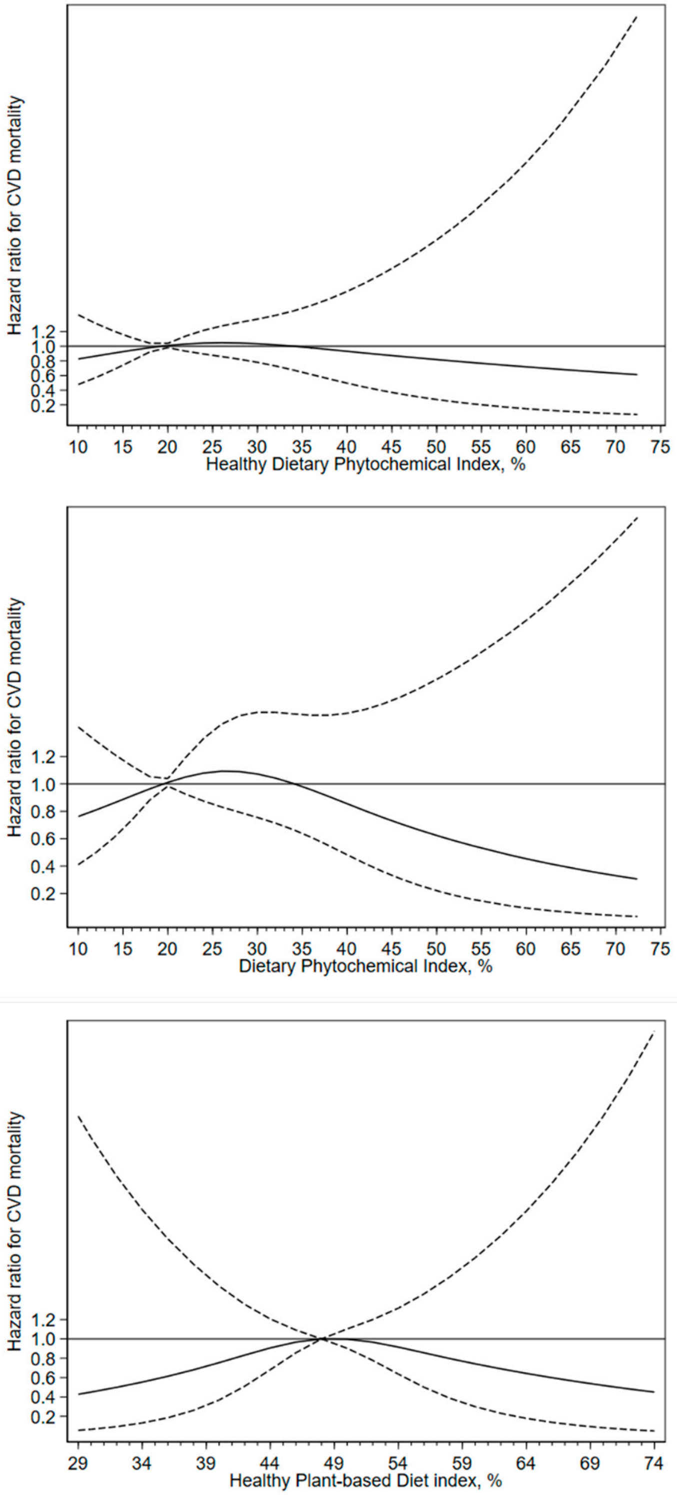

Cubic splines estimated with fully adjusted models: age, sex, educational level, smoking status, alcohol consumption, physical activity, BMI, total caloric intake (only for hPBD), dieting, type 2 diabetes, hypertension, hypercholesterolemia, family history of CVD and family history of Diabetes.
